# Setting research priorities for sexual, reproductive, maternal, newborn, child and adolescent health in humanitarian settings

**DOI:** 10.1186/s13031-021-00353-w

**Published:** 2021-03-26

**Authors:** Loulou Kobeissi, Mahalakshmi Nair, Egmond Samir Evers, Mansuk Daniel Han, Samira Aboubaker, Lale Say, Nigel Rollins, Gary L. Darmstadt, Karl Blanchet, Daniel Martinez Garcia, Olivier Hagon, Per Ashorn, Members of the WHO research prioritization group for SRMNCAH promotion in humanitarian settings, Dina Abbas, Hassan Abdi, Mohannad Al-Nsour, Florence Kamayonza Baingana, Hyam Nicola Bashour, Sara Casey, Primus Che Chi, Henia Dakkak, Delan Devakumar, Michelle Hynes, Sylvia Garry, Josephine Ippe, Nur Jahan Mitu, Sita Anushwan Jojan, Naoko Kozuki, Qamar Mahmood, Philip Mann, Emily Mates, Patrick Onyango Mangen, Elif Özmert, Roberta Petrucci, Hannah Tappis, Gavin Wood

**Affiliations:** 1 SRH Integration in Health Systems (SHS), Department of Sexual and Reproductive Health and Research (SRH), World Health Organization (WHO), Universal Health Coverage – Life Course Division (UHC/LC), Geneva, Switzerland; 2 Department of Maternal, Newborn, Child and Adolescent Health and Ageing (MCA),World Health Organization, Universal Health Coverage – Life Course Division (UHC/LC), Geneva, Switzerland; 3 Independent Consultant, Geneva, Switzerland; 4 Maternal and Child Health, Neonatal and Developmental Medicine, Department of Pediatrics, Stanford University School of Medicine, Stanford, USA; 5 Geneva Centre of Humanitarian Studies, University of Geneva, the Graduate Institute, Geneva, Switzerland; 6 Women and Child Health Unit, Medical Department of Médecins Sans Frontières (MSF), Geneva, Switzerland; 7 Center for Humanitarian Medicine and Disaster Management (CHMDM), WHO Collaborative center, Division of Tropical and Humanitarian Medicine, Geneva University Hospitals, Geneva, Switzerland; 8 Center for Child Health Research, Faculty of Medicine and Health Technology, Tampere University, Tampere, Finland

**Keywords:** Research priorities, CHNRI, Delphi, Sexual health, Reproductive health, Maternal health, Newborn health, Child health, Adolescent health, Humanitarian conflict, Humanitarian pediatrics

## Abstract

**Background:**

An estimated 70.8 million people are forcibly displaced worldwide, 75% of whom are women and children. Prioritizing a global research agenda to inform guidance, service delivery, access to and quality of services is essential to improve the survival and health of women, children and adolescents in humanitarian settings.

**Method:**

A mixed-methods design was adapted from the Child Health and Nutrition Research Initiative (CHNRI) methodology to solicit priority research questions across the sexual, reproductive, maternal, newborn, child and adolescent health (SRMNCAH) domains in humanitarian settings. The first step (CHNRI) involved data collection and scoring of perceived priority questions, using a web-based survey over two rounds (first, to generate the questions and secondly, to score them). Over 1000 stakeholders from across the globe were approached; 177 took part in the first survey and 69 took part in the second. These research questions were prioritized by generating a research prioritization score (RPP) across four dimensions: answerability, program feasibility, public health relevance and equity. A Delphi process of 29 experts followed, where the 50 scored and prioritized CHRNI research questions were shortlisted. The top five questions from the CHNRI scored list for each SRMNCAH domain were voted on, rendering a final list per domain.

**Results:**

A total of 280 questions were generated. Generated questions covered sexual and reproductive health (SRH) (*n* = 90, 32.1%), maternal health (*n* = 75, 26.8%), newborn health (*n* = 42, 15.0%), child health (*n* = 43, 15.4%), and non-SRH aspects of adolescent health (*n* = 31, 11.1%). A shortlist of the top ten prioritized questions for each domain were generated on the basis of the computed RPPs. During the Delphi process, the prioritized questions, based on the CHNRI process, were further refined. Five questions from the shortlist of each of the SRMNCAH domain were formulated, resulting in 25 priority questions across SRMNCAH. For example, one of the prioritized SRH shortlisted and prioritized research question included: “*What are effective strategies to implement good quality comprehensive contraceptive services (long-acting, short-acting and EC) for women and girls in humanitarian settings?”*

**Conclusion:**

Data needs, effective intervention strategies and approaches, as well as greater efficiency and quality during delivery of care in humanitarian settings were prioritized. The findings from this research provide guidance for researchers, program implementers, as well as donor agencies on SRMNCAH research priorities in humanitarian settings. A global research agenda could save the lives of those who are at greatest risk and vulnerability as well as increase opportunities for translation and innovation for SRMNCAH in humanitarian settings.

**Supplementary Information:**

The online version contains supplementary material available at 10.1186/s13031-021-00353-w.

## Background

According to United Nations High Commission for Refugees (UNHCR) in 2019, over 70.8 million people are estimated to be forcibly displaced, of which 25.9 million are refugees. This number has reached its highest point on record and is further complicated with a total of 235 million people in need of humanitarian assistance as estimated by the Global Humanitarian Overview in 2021. 75% of refugees are women and children (of whom, 34 million are adolescent girls and young women) [1].

Humanitarian crises are diverse and range from forced internal displacement, to natural disasters, famine, communicable disease outbreaks, and/or armed conflict. They pose important health implications, given that they are frequently associated with collapsed or severely damaged health systems, including lack of essential medications and contraceptives, absence of skilled health professionals and/or the inability to access them, and overall limited quality of care (inclusive of the absence of blood transfusions and basic surgery procedures) [2, 3]. Further, the world is challenged by the increasing numbers of protracted crises. Currently, the average time spent in displacement is estimated at 25 years, during which transitioning in the provision of care from acute to comprehensive sexual, reproductive, maternal, newborn, child and adolescent health (SRMNCAH) services often fails to occur [2, 3].

The increased number and nature of humanitarian crises pose tangible threats to achieving the Sustainable Development Goals, especially Goal 3 for health, and the attainability of universal health coverage and leaving no one behind. The Global Strategy for Women’s Children’s and Adolescents’ Health (2016–2030) and the World Health Organization’s (WHO’s) 13th Global Programme of Work, emphasize the need to intensify efforts to deliver evidence-based interventions for the health of the world’s most vulnerable people.

Many of the countries with the poorest SRMNCA health indicators are currently or have recently been impacted by a humanitarian crisis [4–6]. Recent figures indicate that maternal and under-five child deaths are highest in countries affected by humanitarian emergencies [7–10]. Moreover, women and adolescent girls face unique vulnerabilities during humanitarian crises, due to increased rates of exposure to sexual and domestic violence [11–13], complications during pregnancy and delivery (i.e. increased rates of induced deliveries and caesarean sections in order to insure a safe delivery), induced septic unsafe abortions, anemia due to food insecurity, as well as increased rates of sexually transmitted and reproductive tract infections including HIV [14, 15].

Significant global gains in SRMNCAH are difficult to achieve without good understanding of barriers, and potential solutions, for the promotion and delivery of services before, during and after crises. For this to be achieved, there is need for guidance on research priorities in humanitarian settings specifically around SRMNCAH promotion and service delivery in conditions and contexts where populations are displaced, live in temporary shelters and/or in adverse environments.

The WHO Departments of Maternal, Newborn, Child and Adolescent Health and Aging (WHO/MCA) and Sexual and Reproductive Health and Research (WHO/SRH) conducted a research prioritization exercise in 2018–2019 to identify a set of global research priorities for improving SRMNCAH in humanitarian settings. The overall goal of this exercise is to guide the global research agenda for better SRMNCAH outcomes in humanitarian settings.

Specifically, WHO/MCA and WHO/SRH aimed for actionable medium-term priorities to generate research and inform guideline development in this area until 2025. In this paper, we describe the methodology of this process, discuss the main findings and recommend a way forward for potential dissemination and scale up.

## Methods

**Fig. 1:**
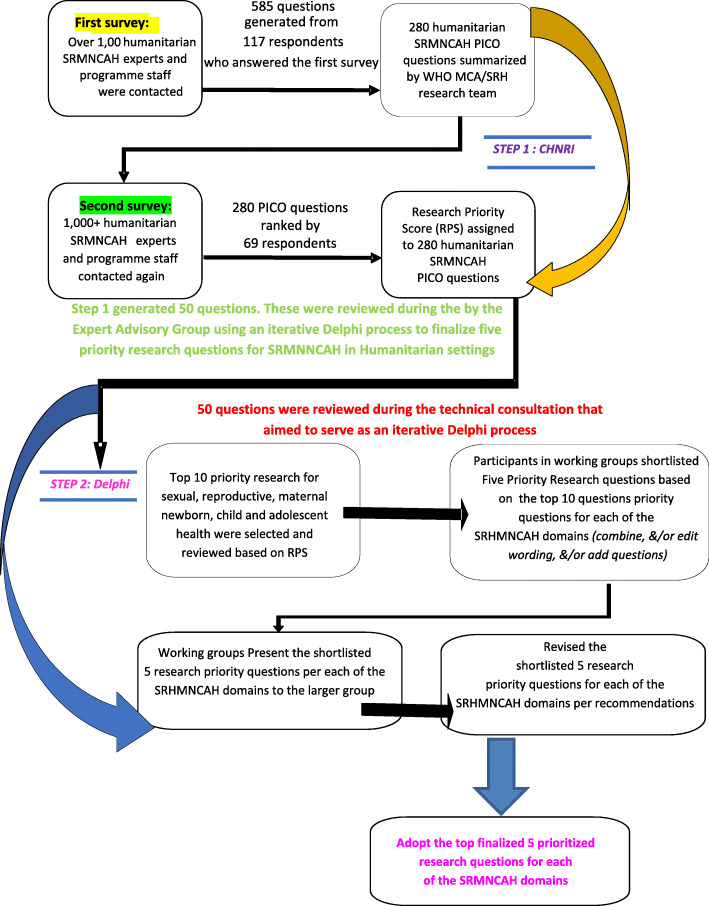
Overview of the CHNRI & Delphi Process

We employed a two-step approach. First, we first gathered what the key stakeholders perceived as priority research questions for improving SRMNCAH in humanitarian settings guided by the Child Health and Nutrition Research Initiative (CHNRI) methodology [16–21]. The CHNRI methodology is a systematic approach of aligning health research investments with the potential impact of research [22–24]. It has been used to identify research priorities and gaps for many topics in SRMNCAH, mental health, and disability [18–21, 25–33]. Then, we established expert consensus on the top research priority questions per SRMNCAH domain among the questions gathered from the CHNRI exercise through a Delphi process (Fig. 1).

### Step 1: CHNRI process

The CHNRI process deployed two web-based surveys: first survey to obtain research questions for improving SRMNCAH in humanitarian settings, and second survey to rank the perceived priorities of the questions identified from the first survey.

Survey participants were identified using multiple methods, such the lists’ of participants of WHO’s past research priority exercises and through snowballing using various SRMNCAH and health emergencies network that represent a diverse spectrum of global geography and organizations. The participants were self-identified by respective expertise in maternal health, SRH, adolescent SRH, newborn health, child health, or adolescent health excluding SRH.

In both surveys, the participants were asked to focus on contextualizing the research questions by type, severity, and duration of emergencies, as well as socio-cultural issues surrounding the context when both proposing and ranking the questions. They were also asked to frame broad research questions to allow for cross-cutting intervention research and mention any known evidence gaps to inform future guideline development. Because the surveys used were part of a research prioritization exercise (as opposed to an actual research project), ethical clearance was not needed. Further, The surveys did not contain or seek any personal identifiers, personal data, and/or any confidential information.

1. First CHNRI survey: stakeholders proposing priority research questions.

The first survey (Supplementary Table A) participants were asked to propose up to five priority research questions, which were initially categorized by the participants into groups for target population, outcome under study, main strategy or focus of the intervention under study, proposed type of research, phase of emergency, and setting. Data cleaning focused on removing clearly inappropriate questions and deduplication. Following which, the questions were further refined into the population, intervention, comparison, and outcome (PICO) formats (Supplementary Table B) for a review by the Technical Advisory Group (Supplementary Table D).

2) Second CHNRI survey: ranking the priority order of questions from the first survey.

A second survey was sent to the respondents of the first survey to rank the PICO-formatted questions. Respondents were given the liberty to rank either only the questions in the domain(s) most relevant to their field of expertise or all questions based on the following four dimensions [21]:

- Answerability: It will be possible to design an ethically sound and implementable research study that can provide the requested answer.
- Program Feasibility: The achieved answer can be translated into a deliverable and affordable public health intervention.
- Public health relevance: The emerging intervention is likely to substantially improve health in the intended target population.
- Equity Value: Answering the question can facilitate interventions that reduce population inequities, i.e. preferentially improve the health of the most vulnerable and disadvantaged.

The respondents were asked to rate the research questions based on these dimensions, ranging from: yes = 1, possible = 0.5, and no or cannot answer = 0. For each question, a research priority score (RPS) was calculated, by taking an arithmetic average of the four average dimension scores for each research question.

Based on the RPS, the top 10 priority research questions for each domain were identified. After an appraisal from the Technical Advisory Group, these questions served as the basis for the second step, i.e. the Delphi process (Supplementary Table D).

### Step 2: Delphi process

The second step, Delphi process, was built on the CHNRI process in Step 1 and focused on consensus building for the research priorities during a technical expert consultation meeting held in Geneva, Switzerland in April 2019. Twenty-nine global experts participated in this exercise to further refine the research questions and build consensus on the top research priorities. The expert group included broad representation across regions, organizations and area of expertise. Notably it also included experts from different humanitarian contexts such as Bangladesh, Jordan, Iraq, Kenya, Nigeria, Syria, Turkey, and Uganda to ensure inclusion of diverse points of view.

The expert group discussed the top 10 SRMNACH research questions in domain sub-groups to formulate top 5 research priority questions per domain. While, there was still the possibility to include new questions, it was agreed that at least 3/5 were to be drawn directly from the top 10 questions identified from the CHNRI process. To ensure that the final results were aligned with the CHNRI process outputs, the group members were requested to refer to the original CHNRI PICO-formatted questions as much as possible, and demonstrate how the questions were strengthened and/or merged as required during plenary discussions.

## Results

Over 1000 stakeholders from diverse contexts were approached, and 177 participants took part in the first survey round of the CHNRI for collecting the possible research questions. A total of 69 experts (Table 1) took part in the scoring exercise during the second survey round of the CHNRI. The majority of the experts who participated in the second survey were child health (*n* = 38) and newborn health experts (*n* = 37). They worked predominantly in research (*n* = 19), at INGOs (n = 19) or with national governments (*n* = 16). There was broad representation of WHO Regions. The main target population for the solicited research questions were pregnant or post-delivery women as well as adolescents.

**Table 1:** Distribution of Experts by Organization, Expertise and Region for the two CHNRNI Surveys (Soliciting *(Survey 1)* and Scoring *(Survey* 2*)*)

Respondents	First Survey (***n*** = 177)	Second Survey (***n*** = 69)
	Number of Respondents (%)	Number of Respondents (%)
***By: Organization Type***		
International NGO	49 (28)	19 (28)
Academic / research	45 (25)	19 (28)
UN agency	30 (17)	3 (4)
National government	25 (14)	16 (23)
National NGO	1 (1)	8 (12)
Other	10 (6)	3 (4)
Regional NGO	3 (2)	0 (0)
Foundation	2 (1)	0 (0)
Industry / enterprise	1 (1)	1 (1)
***By: Expertise***		
Sexual and reproductive health	65 (37)	30 (43)
Maternal health	49 (28)	32 (46)
Newborn health	48 (27)	37 (54)
Child health	58 (33)	38 (55)
Adolescent health	29 (16)	29 (42)
Other	32 (18)	4 (6)
***By: Region***		
AFRO	23 (13)	11 (16)
PAHO	55 (31)	18 (26)
EMRO	21 (12)	9 (13)
EURO	62 (35)	20 (29)
SEARO	12 (7)	8 (12)
WPRO	4 (2)	3 (4)

*NGO* Non-governmental organization

*UN* United Nations

*AFRO* WHO Regional Office for Africa

*PAHO* WHO Regional Office for the Americas

*EMRO* WHO Regional Office for the Eastern Mediterranean

*EURO* WHO Regional Office for Europe

*SEARO* WHO Regional Office for South-East Asia

*WPRO* WHO Regional Office for the Western Pacific

Of the 570 question generated during the first survey round of the CHNRI, 280 were rendered to serve as the final list of questions after cleaning and removal of duplicates (Supplementary Table B). The distribution of response rates for each of the SRMNCAH domains during the second CHRNI survey was as follows: for the SRH domain was 59. 4% (41 out of 69 respondents), for the Maternal health was 59. 4% (41 out of 69 respondents), the Newborn health: 73.9% (51 out of 69 respondents), for the Child health domain was 73.9% (51 out of 69 respondents) and for the Adolescent health (non-SRH) domain was 73.9% (51 out of 69 respondents).

Ninety of the 280 questions (32.1%) covered SRH, and the average RPS for each of the top ten CHNRI-prioritized SRH questions ranged from 0.771 to 0.835. Seventy-five questions (26.8%) covered maternal health (average RPS for the top ten questions: 0.774 to 0.814), and 42 questions (15.0%) covered newborn health (average RPS for the top ten questions 0.779 to 0.873). It should be noted that four questions (1. 4%) covered maternal and newborn health together. Forty-three questions (15. 4%) covered child health (average RPS for the top ten questions 0.721 to 0.794), and 31 questions (11.1%) covered non-SRH adolescent health (average RPS for the top ten questions 0.659 to 0.765) All top-ten CHNRI-prioritized questions per each of the SRMNCAH domains, as described, had high RPS, i.e. closer to 1 (Supplementary Table C).

During Step 2, the Delphi process, 25 questions were adopted after refinements following an iterative process and based on from the CHNRI scored list, i.e. five from each of the five SRMNCAH domains.

The prioritized SRH questions focused on testing: effective strategies for comprehensive family planning use, effective strategies for the integration of mental health/psychosocial support into SRH programming in humanitarian settings as well as capacity building approaches and support mechanisms to strengthening capacity for health workers during SRH service response. The prioritized maternal health research questions focused on: testing surveillance methodologies to capture maternal and perinatal mortality at the population level, effective strategies/approaches (task shifting, self-care, community health workers, mobile clinics, digital technologies) to provide maternal and perinatal health services as well as the impacts of unconditional cash transfers on reducing maternal mortality in humanitarian settings (Table 2).

**Table 2:** Distribution of the Final Prioritized Research Questions for the Sexual, Reproductive and Maternal Health domains following the Delphi process

***Sexual and reproductive health***	
What are effective strategies to implement good quality comprehensive contraceptive services (long-acting, short-acting and EC) for **women and girls** in humanitarian settings?	
What are the effective strategies to improve SRH status (e.g. Reduce teenage pregnancy, increase contraceptive uptake) of **adolescents** in humanitarian settings?	
What are successful strategies to deliver a full range of contraceptives including long-acting methods of contraception from the **onset of a humanitarian** emergency?	
What are best strategies to **integrate** mental health/psychosocial support into SRH programming in humanitarian settings?	
What capacity building approaches and support mechanisms are effective at strengthening capacity for **health workers** for different SRH components in humanitarian settings?	
***Maternal health***	
Which surveillance methodologies/modalities and strategies are most effective to capture and understand maternal and perinatal mortality at the population level?	
What are the most effective strategies/approaches (task shifting, self-care, community health workers, mobile clinics, digital technologies) to provide maternal and perinatal health services (inclusive mental health, nutrition)?	
Are unconditional cash transfers an effective strategy for reducing maternal mortality in humanitarian settings?	
How effective and acceptable are existing interventions and tools (i.e. harm reduction models, task-sharing, self-management, outpatient management) to assist pregnant persons seeking an induced abortion in humanitarian settings?	

*EC* Emergency contraception

*SRH* Sexual and reproductive health

The prioritized newborn health research questions focused on testing: task shifting approaches for intrapartum and immediate postpartum service delivery (home versus PHC), the delivery of essential newborn care in improving newborn outcomes as well as testing different data collection strengthening approaches to monitor newborn mortality for better accountability (Table 3). The prioritized child health research questions focused on testing: whether the integration of inclusive nurturing care for early childhood development will promote greater health and development for children, the effectiveness of community-based management approaches in reducing morbidity and mortality for children under five in humanitarian settings, whether the current delivery of nutrition interventions in refugee camps meets the needs of high-risk infants and children such as pre-terms, or low birth weight infants or infants with perinatal injury as well as the impacts of the demographic, social and operational factors on incomplete childhood vaccination in conflict-affected populations. The prioritized adolescent health (non SRH) research questions focused on exploring and understanding: the drivers of mental health disorders, substance use and risky behaviour amongst adolescents who have been forced to migrate, the pathways needed to facilitate access of GBV survivors to the appropriate support in humanitarian contexts, the effectiveness of different nutritional interventions in improving functional outcomes (cognitive, physical, etc.) among adolescents as well as the effectiveness of facilitated and/or peer-led groups in addressing the psychosocial needs for adolescents in humanitarian settings (Table 4).

**Table 3:** Distribution of the Final Prioritized Research Questions for the Newborn Health domain following the Delphi process

***Newborn health***	
In acute and protracted conflict-affected contexts (*to specify), what are the most effective models to task shift intrapartum and immediate postpartum service delivery (as pertinent ENC / BEmONC) to the home or to primary health centers?	
In acute and protracted conflict-affected contexts (*to specify), what are the most effective models to deliver essential newborn care?	
In acute and protracted conflict-affected contexts (*to specify), what are the most effective models to provide pregnancy / newborn care education to relevant caregivers?	
In acute and protracted conflict-affected contexts (*to specify), what are the most effective models to care for vulnerable newborns (small and sick)?	
In acute and protracted conflict-affected contexts (*to specify), what are the most effective models to collect, interpret, and act on valid mortality data (including stillbirths)?	

*ENC* Essential newborn care

*BEmONC* Basic emergency obstetric and newborn care

**Table 4:** Distribution of the Final Prioritized Research Questions for the Child and Adolescent Health domains following the Delphi process

***Child health***	
Does identification and management of nutritionally at-risk infants aged < 6 months reduce morbidity and improve infant growth/development in humanitarian settings? **And HOW?**	
Is community-based management an effective approach for reducing morbidity and mortality among under five-year-old children humanitarian settings?	
How do current nutrition interventions delivered in refugee camps meet the needs of high-risk infants/children, such as those born preterm, low birth weight, or with perinatal injury?	
What are the demographic, social and **operational** factors that are associated with incomplete childhood vaccination status in conflict-affected populations?	
Does the integration of group health and nutrition promotion with infant stimulation and play in a safe space within the community, lead to a better wellbeing of the mother and social and cognitive development of children OR does the provision of organised integrated and inclusive nurturing care for early childhood development through the health services during protracted emergencies promote **GREATER** health and development of children?	
***Adolescent health (Non SRH)***	
What are the drivers of mental health disorders, substance use and risky behaviour amongst adolescents who have been forced to migrate?	
What social and mental health interventions are effective in reducing the negative consequences of forced child/early marriage of adolescents in humanitarian settings?	
What pathways need to be available for GBV survivors to access appropriate support in humanitarian settings?	
What nutritional interventions are effective in improving functional outcomes (cognitive, physical, etc.) in adolescents in humanitarian settings?	
What is the effectiveness of facilitated and/or peer-led groups on assessing and addressing the psychosocial needs for adolescents in humanitarian settings?	

*SRH* Sexual and reproductive health

*GBV* Gender-based violence

## Discussion

Funding for public health research in complex humanitarian settings is limited, more so than in many other settings [27], in part due to contextual barriers that hamper the ability to undertake research in these settings. The CHNRI methodology allows us to systematically align health research investments with the potential impact of research, by convening key stakeholders to identify priority research questions [22–24]. Using a CHNRI process, followed by a Delphi process, we were able to set research priorities for SRMNCAH in humanitarian settings, with a final list of top 25 priority research questions for SRMNCAH in humanitarian settings.

We believe that the agreed upon top 25 SRMNCAH research priority questions will contribute to guiding the global health agenda for SRMNCAH research in humanitarian settings for the coming five years and beyond (i.e. to 2025). Many of these prioritized research questions address key gaps in current knowledge around SRMNCAH in humanitarian settings as pertinent to: needs, availability and quality of existent services; impact of implemented technical as well as operational guidance, as well as over-arching outcomes and impacts of humanitarian response. Hence, in many ways, these identified research priorities can help address these key gaps and accelerate progress. Particularly, these research questions focused on the need to improve data and surveillance systems, test integrative approaches to care and test new intervention strategies for delivery of care across the SRMNCAH domains. These findings reflect the current realities of the humanitarian field, which revolves around scarcity of data to guide preparedness, response and recovery, as well as the need for implementation research to inform the effective delivery of essential SRMNCAH interventions in various humanitarian settings. They also bring about novel priority questions, some of which include: exploring the effectiveness and impacts of cash-transfers on maternal mortality (in many developing countries, and particularly those with humanitarian crises, cash transfers can be highly controversial); exploring the impacts of enabling preventive services (such as contraception, ANC, use of skilled birth attendants, availability of emergency obstetric care and referral system failures) on maternal mortality; as well as identifying determinants to strengthen surveillance as well as data and monitoring in humanitarian settings beyond maternal morbidity and mortality. It merits to note as well that some of the prioritized research questions extended beyond crisis/humanitarian settings. The generated PICO questions were cleaned and refined to the best extent possible, however, retaining the fidelity of the original solicited questions by the survey respondents is critical to the internal validity of this exercise. For this reason, some research questions could have been missed out because they were not perceived as a priority for the different stakeholders who participated in this research prioritization exercise.

Here, we further strengthened the trustworthiness of the CHNRI findings by introducing a follow up Delphi process to achieve an additional layer of prioritization. Although, similar research was conducted using the CHNRI methodology on neonatal health in complex humanitarian settings in 2014 [21], our exercise is the first to provide a global list of SRMNCAH research priorities in humanitarian settings.

Although, the CHNRI methodology offers many advantages, there are still numerous limitations. Given the low response rate that we had during the first and the second round of surveys, there may be additional questions that were missed that might warrant additional consideration. Further, these results could represent a biased opinion of experts, i.e. opinion is only limited to those experts who participated in the surveys, which could limit the external validity of the results.

During both the CHNRI and the Delphi processes, extensive efforts were made to reach a broad and diverse range of audience, including humanitarian workers in the field, government public health officials in affected contexts, NGOs and INGOs engaged in providing humanitarian assistance, UN organizations, academic institutions involved in researching humanitarian contexts and collaborative networks. While various routes were utilized for wide dissemination, participation may have been influenced by the English language questionnaire, the technical subject matter and the snowballing approach that could have targeted better “networked” stakeholders.

Nevertheless, we remain confident in the results of this exercise because of the number and the diversity of experienced global experts who participated in this exercise, given their significant expertise and knowledge on SRMNCAH in humanitarian settings and ability to judge a very diverse spectrum of research questions. Also, the fact that we coupled the CHNRI with a Delphi process on one hand, and having an independent technical advisory group, on the other hand, to oversee the different stages of this exercise, allowed to validate the findings reported here. The participation rates observed in our study are very similar to those reported elsewhere for other CHNRI research priority exercises [19–21, 25–33].

We believe that this collaborative research prioritization exercise will help enhance and promote trust amongst the various key stakeholders responsive for SRMNCAH in humanitarian settings, by stimulating shared interests, advocacy, and continued engagements. The here-in prioritized SRMNCAH questions could serve as a guiding research map for SRMNCAH in humanitarian settings and simultaneously could increase opportunities for translation and innovation. It is the proposition in this paper that when these priority SRMNCAH research questions become translated into collaborative research activities across and throughout the globe, with a focused goal of translating research into action, that these prioritized questions become more meaningful, human centered, as well as provide evidence-driven solutions to effectively respond to the contextual humanitarian settings’ needs. This, ultimately, results in developing and supporting a long relationship building process amongst the different and key stakeholders in the field from donors, researchers, national governments, providers and most importantly the end users.

## Conclusion

To be able to cater to the complex realities of humanitarian settings, a comprehensive and global research agenda [that could fill in gaps and caveats in data needs, effective intervention strategies and approaches, as well as inform better efficiency and quality during delivery of care in humanitarian settings] is a must. We combined a CHNRI and a Delphi to identify a set of research priorities for SRMNCAH in humanitarian settings. These research questions can inform and contribute to our understanding of priorities and delivery mechanisms for SRMNCAH in humanitarian settings, with the ultimate aim to improve the survival and health of the population in those settings. Moving this forward, WHO MCA and SRH departments aim for actionable medium-term priorities to inform and guide research in this area until 2025, in order to increase opportunities for translation and innovation for SRMNCAH in humanitarian settings.

This will entail follow up with all relevant stakeholders who participated in this exercise and beyond in order to scale up the findings of this exercise as well as to allow for advocating and setting up the needed partnerships, collaborations and funding for as many as possible of the prioritized SRMNCAH research questions in humanitarian settings. Some of the research consortia, partnerships and collaborations that can engaged in this process, could include: Global Health Cluster; BRANCH Consortium, Inter-Agency Working Group on Reproductive Health in Crises (IAWG), Partnership for Maternal, Newborn, and Child Health (PMNCH), A Network for Improving Quality of Care for Maternal, Newborn and Child Health (QOC Network), Every Mother Every Newborn (EMEN), International Paediatric Association (IPA), Early Childhood Development Action Network (ECDAN), etc.

## Supplementary Information

**Additional file 1: Supplementary Table A**: Research Question Generation Survey (Print Screens of Online Survey). **Supplementary Table B**: Top Research Priority Questions Solicited (*n* = 280). **Supplementary Table C**. Distribution of RPS scores per dimension for the top TEN CHNRI research priority questions per SRMNCAH domain. **Supplementary Table D**. Members, Institutional Affiliations and Terms of Reference of the Technical Advisory Group.

**Additional file 2: Supplementary Material 1**. Interview Guide. **Supplementary Material 2**. **Table 1**. Quality criteria for reporting qualitative research (COREQ) and **Table 1**. Domains, items, description, information in the study.

## Data Availability

Available upon request.
