# Supplementary material for: Setting research priorities for sexual, reproductive, maternal, newborn, child and adolescent health in humanitarian settings
Source: Confl Health. 2021 Mar 26;15:16. doi: 10.1186/s13031-021-00353-w (PMC7995567; doi:10.1186/s13031-021-00353-w)
Supplement: Supplementary file 1 — Additional file 1: Supplementary Table A: Research Question Generation Survey (Print Screens of Online Survey). Supplementary Table B: Top Research Priority Questions Solicited (n = 280). Supplementary Table C. Distribution of RPS scores per dimension for the top TEN CHNRI research priority questions per SRMNCAH domain. Supplementary Table D. Members, Institutional Affiliations and Terms of Reference of the Technical Advisory Group. [file 13031_2021_353_MOESM1_ESM.docx]

**Supplementary Table A: Research Question Generation Survey**

**(Print Screens of Online Survey)**


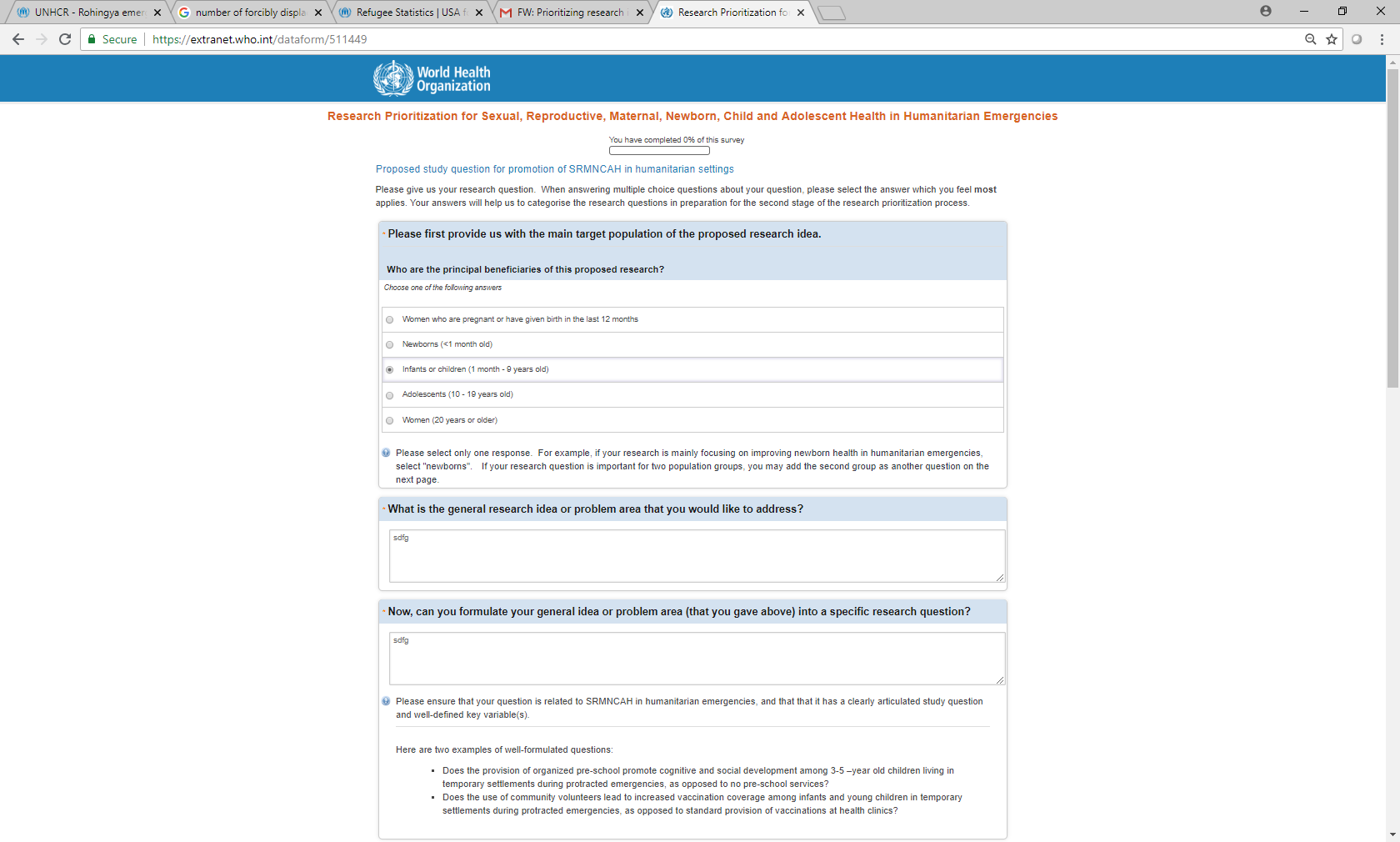

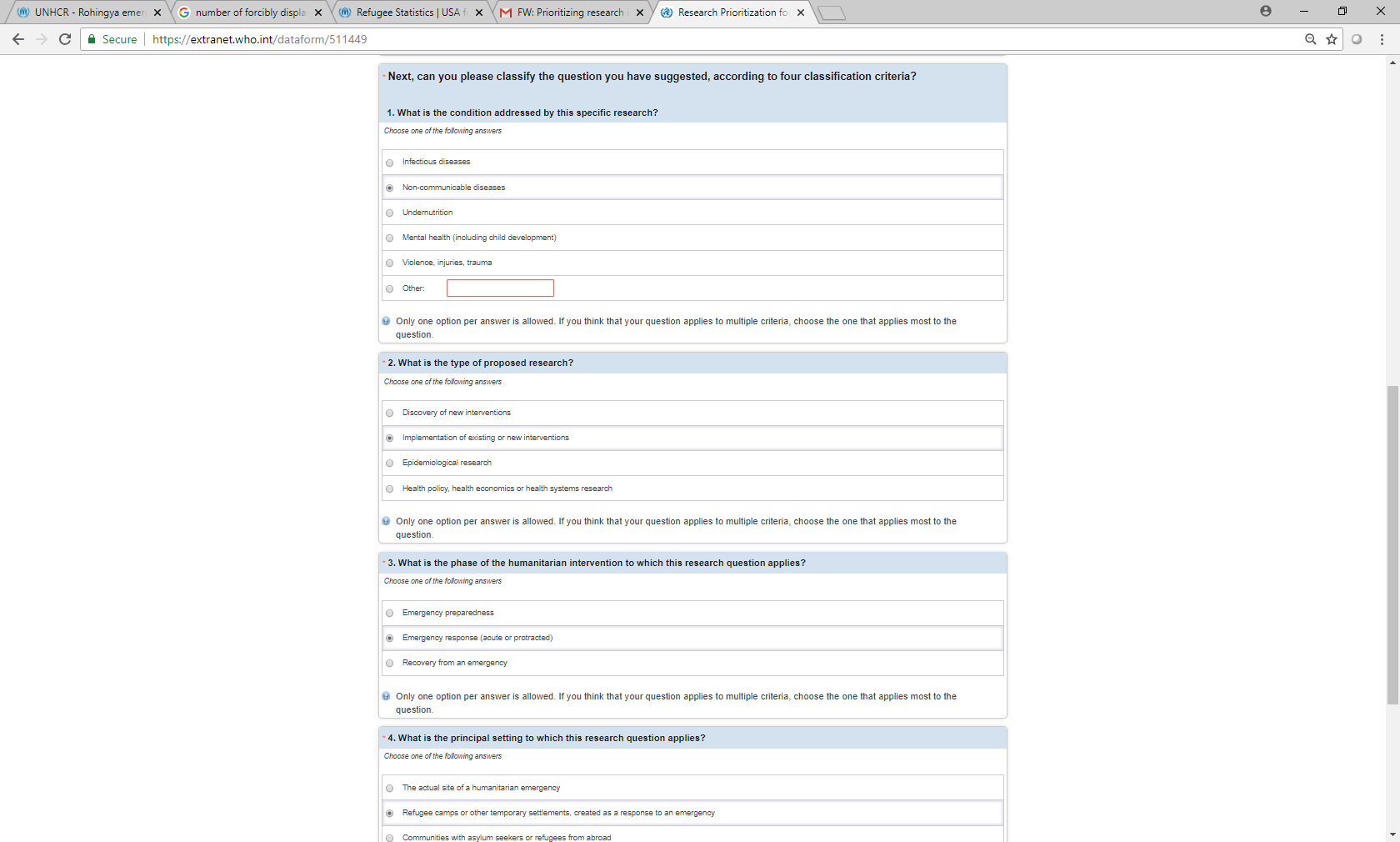


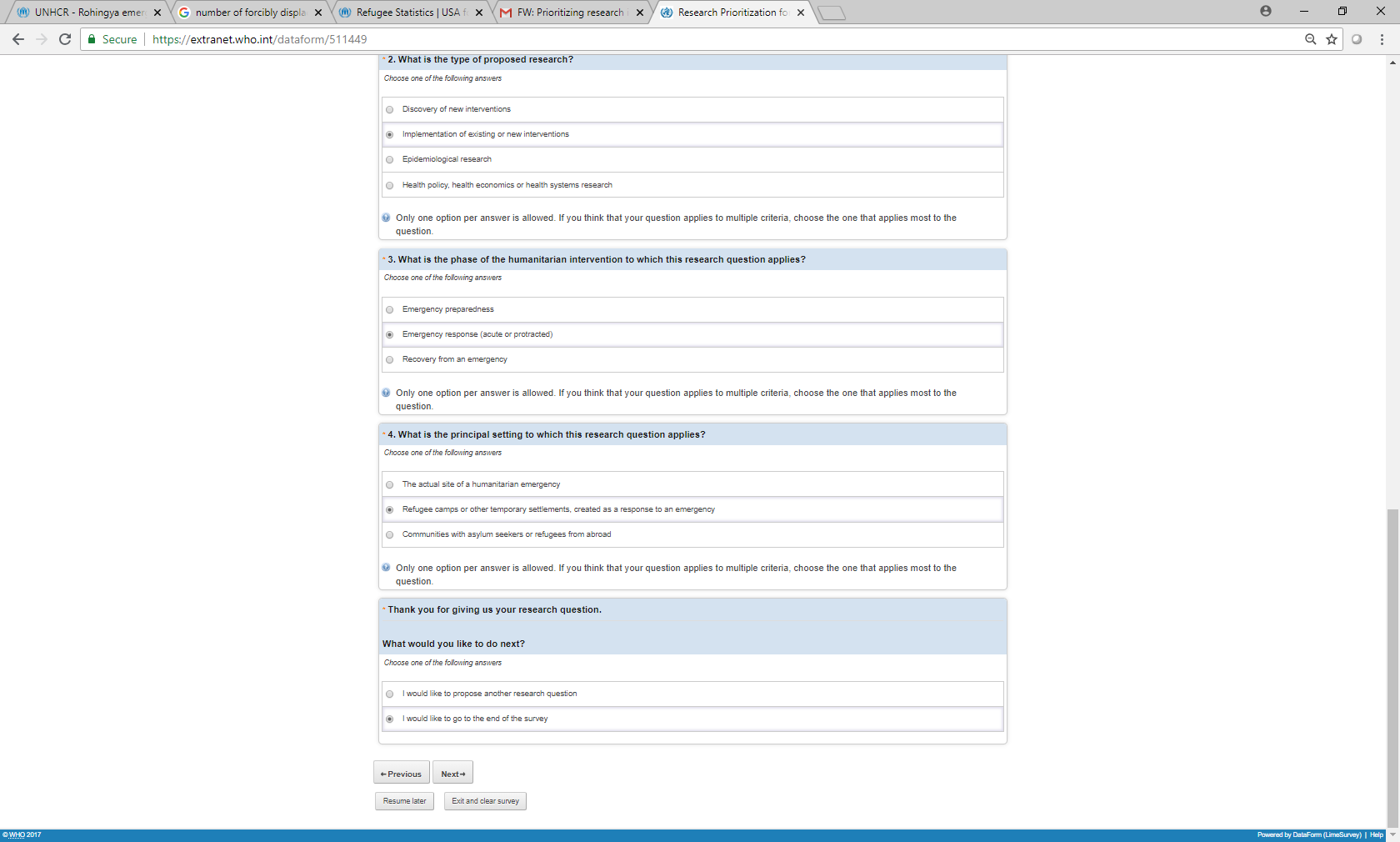


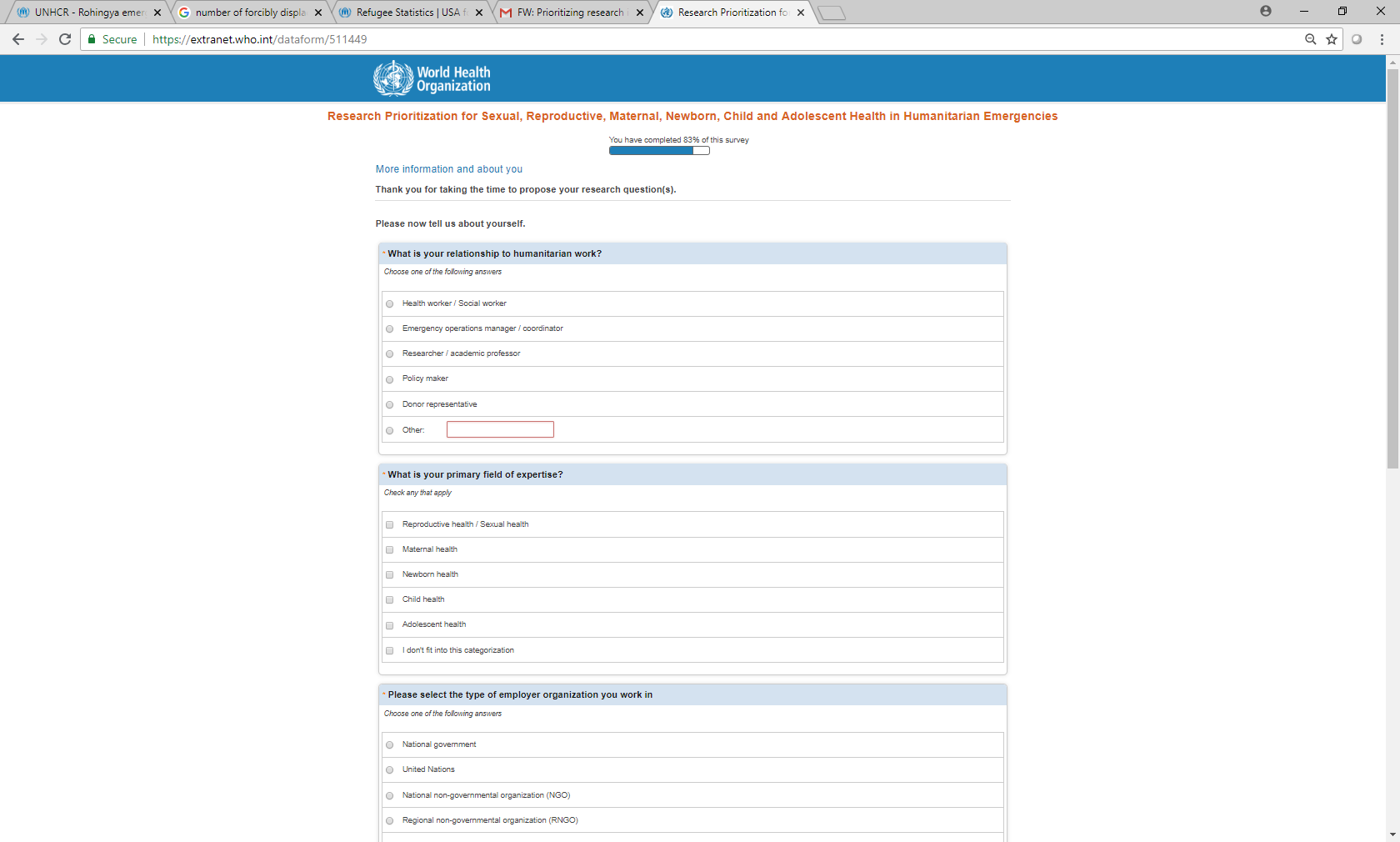

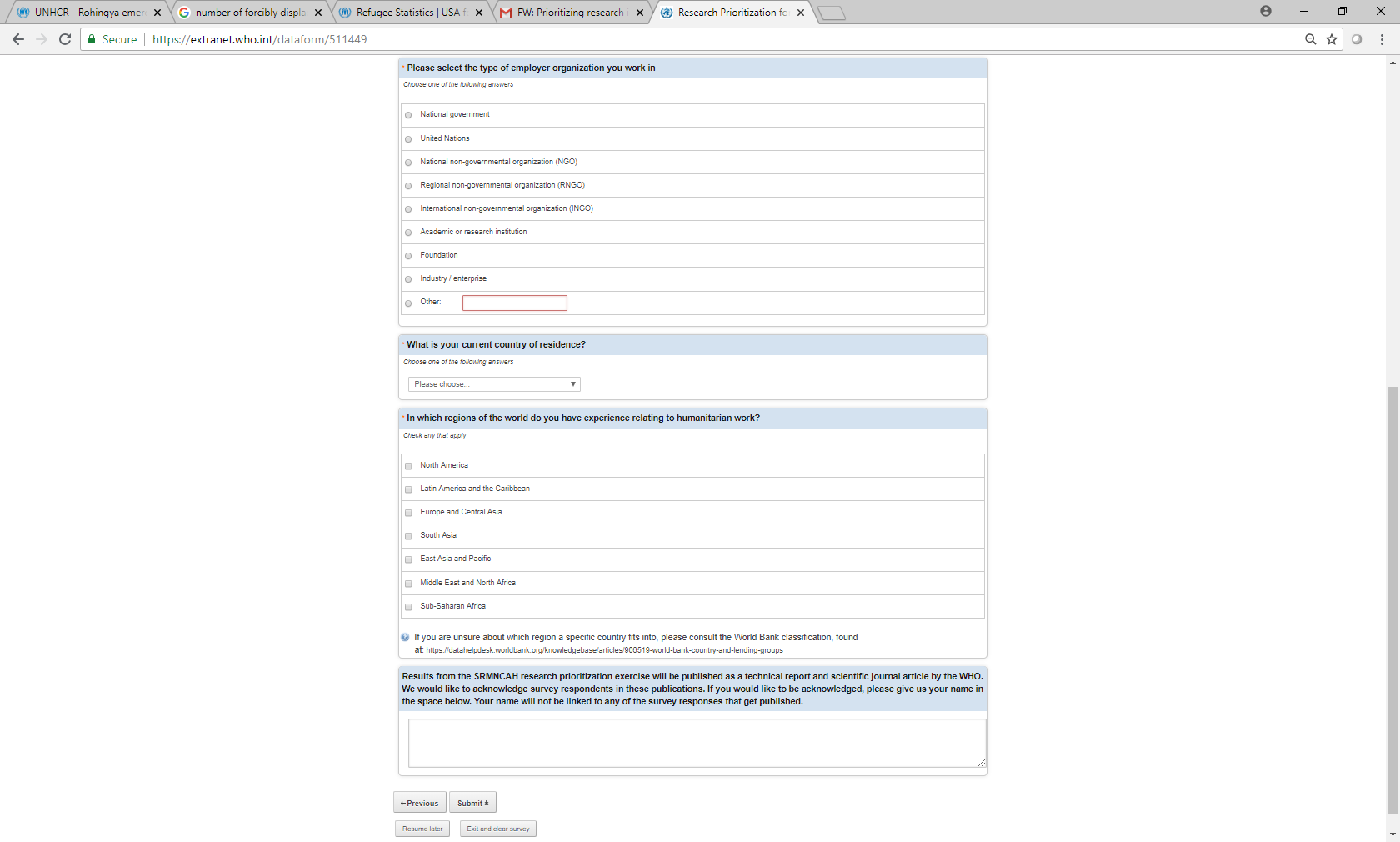


**Supplementary Table B: Top Research Priority Questions Solicited (n=280)**

| **RPS Ranking** | **Question (* = duplicates)** | **Theme** | **Approach** | **Addressed Condition** | **Average RPS** | **Answerable** | **Programme feasible** | **Public health relevant** | **Equity value** | **# of respondents** |
| --- | --- | --- | --- | --- | --- | --- | --- | --- | --- | --- |
| **1** | What are the common causes of newborn mortality and morbidity in different types of humanitarian emergency? | Newborn health | Background information | Crosscutting | 0.873 | 0.902 | 0.833 | 0.902 | 0.853 | 51 |
| **2** | Does the implementation of a home-based hygiene promotion program lead to a reduction of neonatal infections? | Newborn health | Health promotion | Infectious disease | 0.846 | 0.833 | 0.804 | 0.882 | 0.863 | 51 |
| **3** | What are the main causes and underlying determinants of perinatal death in varying humanitarian contexts? | Newborn health | Background information | Crosscutting | 0.841 | 0.824 | 0.794 | 0.912 | 0.833 | 51 |
| **4** | What are the barriers for adolescents' contraceptive use in humanitarian settings? | Sexual and reproductive health | Background information | Contraception | 0.835 | 0.841 | 0.805 | 0.854 | 0.841 | 41 |
| **5** | What are barriers to contraceptive use in humanitarian settings and successful strategies to overcome them? | Sexual and reproductive health | Health system strengthening | Contraception | 0.835 | 0.817 | 0.829 | 0.854 | 0.841 | 41 |
| **6** | Does the community-based distribution of contraceptives to sexually active adolescents contribute to increased prevention of unwanted pregnancies in humanitarian settings, as opposed to the standard provision of contraceptives in the health clinics? | Sexual and reproductive health | Health system strengthening | SRH | 0.829 | 0.829 | 0.817 | 0.841 | 0.829 | 41 |
| **7** | In emergencies, can Kangaroo mother care (KMC) provide as good health outcomes for preterm babies as incubator care? | Newborn health | Health system strengthening | Crosscutting | 0.824 | 0.843 | 0.843 | 0.833 | 0.775 | 51 |
| **8** | Does the provision of adolescent-friendly family planning services in protracted humanitarian emergencies improve their use by 10-19 -year old adolescents, as opposed to standard provision of family planning services? | Sexual and reproductive health | Health system strengthening | SRH | 0.820 | 0.805 | 0.805 | 0.854 | 0.817 | 41 |
| **9** | Is community-based distribution of family planning services, including injectables, applicable and feasible in humanitarian settings and does it enhance people’s access to and use of contraceptives? | Sexual and reproductive health | Health system strengthening | Contraception | 0.817 | 0.866 | 0.805 | 0.817 | 0.780 | 41 |
| **10** | Does economic and knowledge empowerment of adolescent girls and young women reduce the prevalence of teenage pregnancies in refugee settlement camps? | Sexual and reproductive health | Health promotion | SRH | 0.814 | 0.768 | 0.805 | 0.866 | 0.817 | 41 |
| **11** | Does having trained CHWs improve adherence to ANC/PNC and assisted deliveries within displaced populations and to provide better infant care (e.g. exclusive breast feeding, infant stimulation etc.)? | Maternal health | Health system strengthening | MNCAH | 0.814 | 0.805 | 0.805 | 0.829 | 0.817 | 41 |
| **12** | Does a bundle of critical aseptic practices adopted by healthcare providers and caregivers around the time of birth and at home during postnatal care, lead to lower incidence of infections and infection-related neonatal mortality, as compared to, usual care ? | Newborn health | Health promotion | Infectious disease | 0.814 | 0.794 | 0.784 | 0.853 | 0.824 | 51 |
| **13** | In settlement camps, does counseling of parents about home care for newborns and identification of danger signs before discharge reduce early neonatal mortality? | Newborn health | Health promotion | Crosscutting | 0.814 | 0.814 | 0.833 | 0.824 | 0.784 | 51 |
| **14** | For babies born in health centres at low-income country refugee camps, is newborn mortality and morbidity less frequent if the attending midwives/TBAs have been trained in specific delivery room procedures than if the health personell have not been trained in these skills? | Newborn health | Health promotion | Crosscutting | 0.809 | 0.824 | 0.824 | 0.814 | 0.775 | 51 |
| **15** | Does the provision of training to health workers on WHO recommendations for women-centered care for antenatal care, childbirth and post-natal care improve women's satisfaction with health services, childbirth outcomes (including types and nature of interventions during childbirth, outcomes for newborns) and post-natal health (including maternal morbidity and mental health)? | Maternal health | Health system strengthening | MNCAH | 0.805 | 0.841 | 0.817 | 0.793 | 0.768 | 41 |
| **16** | What are effective and cost-effective strategies for maintaining and strengthening capacity of skilled birth attendants to detect and manage obstetric and newborn complications in humanitarian settings? | Maternal health | Health system strengthening | MNCAH | 0.805 | 0.793 | 0.780 | 0.841 | 0.805 | 41 |
| **17** | In an acute phase emergency, which surveillance methodology is most efficient and effective to capture maternal and perinatal mortality at the community level? | Maternal health | Background information | MNCAH | 0.802 | 0.793 | 0.744 | 0.854 | 0.817 | 41 |
| **18** | What barriers do unaccompanied adolescents face in accessing to SRHR information and services as compared to those accompanied by others? | Sexual and reproductive health | Background information | SRH | 0.799 | 0.793 | 0.793 | 0.793 | 0.817 | 41 |
| **19** | Among women in humanitarian crisis contexts, can participation in peer-group sessions during pregnancy increase knowledge about newborn care and improve newborn health outcomes. | Newborn health | Health promotion | Crosscutting | 0.797 | 0.794 | 0.765 | 0.853 | 0.775 | 51 |
| **20** | Does identification and management of nutritionally at-risk infants aged younger than 6 months reduce morbidity and improve infant growth/development in emergency settings? | Child health | Health promotion | Crosscutting | 0.794 | 0.833 | 0.735 | 0.824 | 0.784 | 51 |
| **21** | What are the factors that constrain the utilization of reproductive maternal child and adolescent health (RMNCAH) services by pregnant women in a humanitarian setting? | Maternal health | Health system strengthening | MNCAH | 0.793 | 0.793 | 0.793 | 0.817 | 0.768 | 41 |
| **22** | Is community-based management an effective approach for reducing morbidity and mortality from severe acute malnutrition among under five-year-old children humanitarian settings? | Child health | Care of a sick individual | Undernutrition | 0.792 | 0.814 | 0.784 | 0.794 | 0.775 | 51 |
| **23** | What are effective strategies to deliver proven SRH interventions to adolescents in crises settings? | Sexual and reproductive health | Health system strengthening | SRH | 0.790 | 0.768 | 0.793 | 0.805 | 0.793 | 41 |
| **24** | What are successful strategies to deliver a full range of contraceptives including long acting methods of contraception from the onset of a humanitarian emergency? | Sexual and reproductive health | Health system strengthening | Contraception | 0.790 | 0.744 | 0.805 | 0.817 | 0.793 | 41 |
| **25** | Can mobile health services for providing post natal care to newborns and mothers after a natural disaster decrease mortality and morbidity? | Newborn health | Health system strengthening | Infectious disease | 0.789 | 0.814 | 0.765 | 0.804 | 0.775 | 51 |
| **26** | In refugee camps and other temporary settlements, does a targeted hygiene awareness campaign, including provision of self-hygiene materials (soap, information sheets) reduce the incidence of fecal-oral transmitted diseases among children? | Child health | Health promotion | Infectious disease | 0.789 | 0.814 | 0.804 | 0.784 | 0.755 | 51 |
| **27** | Is the provision of home visits by a skilled attendant to pregnant and postpartum women living in temporary settlements during emergencies more effective, safer and less costly than the use of available standard health facilities? | Maternal health | Health system strengthening | MNCAH | 0.784 | 0.793 | 0.780 | 0.793 | 0.768 | 41 |
| **28** | Do the mobile clinics in protracted emergencies lead to adequate antenatal care coverage among displaced people? | Maternal health | Health system strengthening | MNCAH | 0.780 | 0.805 | 0.768 | 0.780 | 0.768 | 41 |
| **29** | Can existing lifesaving interventions to care for small and sick newborns be adapted to be applicable and practical in a humanitarian context? | Newborn health | Care of a sick individual | Crosscutting | 0.779 | 0.784 | 0.735 | 0.804 | 0.794 | 51 |
| **30** | Does the effective integration of community based newborn care and follow up into health responses during humanitarian emergencies reduce mortality during the first 45 days of life? | Newborn health | Health system strengthening | Crosscutting | 0.777 | 0.775 | 0.735 | 0.824 | 0.775 | 51 |
| **31** | How can maternal mental health care be integrated into pre-conceptional, antenatal, delivery, and post-partum care in humanitarian settings? What are acceptable, effective and cost-effective strategies? | Maternal health | Health system strengthening | Mental health | 0.774 | 0.768 | 0.744 | 0.793 | 0.793 | 41 |
| **32** | Does the provision of women's health services, specifically maternal health services, by skilled providers from the population of displaced persons serve as an effective care model? | Maternal health | Health system strengthening | MNCAH | 0.774 | 0.768 | 0.744 | 0.780 | 0.805 | 41 |
| **33** | What tools are available to assist women experiencing pregnancy loss (inclusive of miscarriage, still birth and neonatal death) in humanitarian settings? | Maternal health | Health system strengthening | MNCAH | 0.774 | 0.793 | 0.768 | 0.768 | 0.768 | 41 |
| **34** | What are the most feasible, effective and efficient methods for measurement or estimation of maternal and perinatal mortality in humanitarian settings? | Maternal health | Health system strengthening | MNCAH | 0.774 | 0.732 | 0.732 | 0.817 | 0.817 | 41 |
| **35** | What are the needs/available resources/attitudes of girls and women to manage menstruation during complex humanitarian emergencies? | Sexual and reproductive health | Background information | SRH | 0.771 | 0.793 | 0.780 | 0.744 | 0.768 | 41 |
| **36** | Does the provision of separate youth health and reproductive health services in humanitarian emergencies increase the uptake of health service use and reduce the frequency and complications of early/unwanted pregnancies (as opposed to provision of services to the general population)? | Sexual and reproductive health | Health system strengthening | SRH | 0.771 | 0.768 | 0.732 | 0.829 | 0.756 | 41 |
| **37** | Does the provision of long acting reversible contraception lead to a decrease in pregnancy rates in women in temporary settlements during protracted emergencies? | Sexual and reproductive health | Health system strengthening | Contraception | 0.771 | 0.805 | 0.817 | 0.732 | 0.732 | 41 |
| **38** | Does improvement in day-of-birth care for pregnant women lead to a reduction in perinatal mortality and morbidity in humanitarian setting? | Newborn health | Health promotion | Crosscutting | 0.770 | 0.775 | 0.716 | 0.804 | 0.784 | 51 |
| **39** | What should be prepositioned in terms of MNCH commodities as preparedness for a crisis? | Maternal health | Health system strengthening | MNCAH | 0.768 | 0.793 | 0.793 | 0.768 | 0.720 | 41 |
| **40** | Does the community-based distribution of condoms contribute to increased prevention of sexually transmitted infections in humanitarian settings, as opposed to the standard provision of condoms in the health clinics? | Sexual and reproductive health | Health system strengthening | STD | 0.768 | 0.780 | 0.780 | 0.756 | 0.756 | 41 |
| **41** | In different kinds of humanitarian settings, can the use of community health workers improve the quality, continuity, and user access to child health services, and reduce costs? | Child health | Health system strengthening | Infectious disease | 0.767 | 0.765 | 0.735 | 0.775 | 0.794 | 51 |
| **42** | How does community health services provide a follow-up of women during the early post partum period who have been discharged from hospital during protracted emergencies? | Maternal health | Health system strengthening | MNCAH | 0.765 | 0.780 | 0.756 | 0.780 | 0.744 | 41 |
| **43** | What is the prevalence and drivers of child marriage in different humanitarian contexts? | Adolescent health | Background information | Child marriage | 0.765 | 0.768 | 0.744 | 0.768 | 0.780 | 41 |
| **44** | What are the challenges and successes in adapting approaches, tools, and guidelines for supply chain management from the development sector and applying them across different humanitarian settings? | General | Health system strengthening | Needs/Challenges | 0.765 | 0.75609756 | 0.75609756 | ######## | ####### | 41 |
| **45** | How do current nutrition interventions delivered in refugee camps meet the needs of high-risk infants/children, such as those born preterm, low birth weight, or with perinatal injury? | Child health | Health system strengthening | Undernutrition | 0.765 | 0.775 | 0.735 | 0.784 | 0.765 | 51 |
| **46** | What is the prevalence of mental health disorders among refugee adolescents in settlement camps? | Adolescent health | Background information | Mental health | 0.765 | 0.784 | 0.755 | 0.784 | 0.735 | 51 |
| **47** | Can community midwives be trained to perform immediate resuscitation for asphyxiated newborns in humanitarian settings? | Newborn health | Health system strengthening | Non-communicable disease | 0.762 | 0.824 | 0.804 | 0.725 | 0.696 | 51 |
| **48** | *What factors determine the use of post-partum family planning services in refugee settings? | Sexual and reproductive health | Background information | Contraception | 0.762 | 0.775 | 0.745 | 0.775 | 0.755 | 51 |
| **49** | What are the Sexual and Reproductive Health and Rights-related needs and challenges of adolescents as well as the situation of service delivery about these needs in humanitarian emergencies? | Sexual and reproductive health | Background information | SRH | 0.762 | 0.768 | 0.732 | 0.780 | 0.768 | 41 |
| **50** | What are the demographic, social and other factors that are associated with complete childhood vaccination status in conflict-affected countries? | Child health | Health promotion | Infectious disease | 0.757 | 0.775 | 0.686 | 0.804 | 0.765 | 51 |
| **51** | How can the SRH services be better integrated with other services? | Sexual and reproductive health | Health system strengthening | SRH | 0.756 | 0.768 | 0.720 | 0.780 | 0.756 | 41 |
| **52** | Are peer to peer support systems an effective way to ensure access and quality of care for pregnancy, delivery, postnatal and family planning services in emergency settings? And what are the key factors contributing to its success? | Sexual and reproductive health | Health system strengthening | Crosscutting | 0.756 | 0.780 | 0.744 | 0.756 | 0.744 | 41 |
| **53** | What are the most effective strategies and policies to retain and sustain the availability and ensure motivation of qualified health workers who provide obstetric and neonatal care in the areas of war and conflict? | Maternal health | Health system strengthening | MNCAH | 0.753 | 0.732 | 0.744 | 0.793 | 0.744 | 41 |
| **54** | What is the view of adolescents affected by disasters about the approaches (sites, providers, timing, etc.) to support their SRH needs? | Sexual and reproductive health | Background information | SRH | 0.753 | 0.805 | 0.720 | 0.732 | 0.756 | 41 |
| **55** | *What are the contraceptive needs, preferences and service coverage of women aged 15-49 after 12 months stay in a humanitarian setting? | Sexual and reproductive health | Background information | Contraception | 0.752 | 0.765 | 0.725 | 0.775 | 0.745 | 51 |
| **56** | What are the underlying social cultural beliefs, practices in humanitarian emergency situations that influence perceptions/agency of young girls 10-19 towards contraceptive services uptake? | Sexual and reproductive health | Background information | Contraception | 0.750 | 0.756 | 0.720 | 0.768 | 0.756 | 41 |
| **57** | Does the use of trained and paid community health workers (CHWs) increase the uptake of family planning and other SRMNCAH services compared to provision in the standard way? | Sexual and reproductive health | Health system strengthening | Contraception | 0.750 | 0.780 | 0.756 | 0.756 | 0.707 | 41 |
| **58** | What services are available to identify and treat maternal mental health conditions, including postpartum depression, in humanitarian emergencies, and are they effective in improving long-term maternal mental health? | Maternal health | Health system strengthening | Mental health | 0.750 | 0.768 | 0.732 | 0.756 | 0.744 | 41 |
| **59** | What are the best ways of ensuring quality midwifery care in acute and protracted crises and ensuring no one is left behind? | Maternal health | Health system strengthening | MNCAH | 0.750 | 0.732 | 0.744 | 0.768 | 0.756 | 41 |
| **60** | Does routine oral cholera vaccination in humanitarian emergencies decrease the burden of acute watery diarrhea in children? | Child health | Health promotion | Infectious disease | 0.748 | 0.784 | 0.735 | 0.745 | 0.725 | 51 |
| **61** | Do community health workers continue to provide curative services during acute crises? | Child health | Health system strengthening | Infectious disease | 0.748 | 0.755 | 0.716 | 0.775 | 0.745 | 51 |
| **62** | What are successful strategies and practical innovations to overcome the barriers for adolescents' contraceptive use and increase the uptake of contraceptives in humanitarian settings? | Sexual and reproductive health | Health system strengthening | Contraception | 0.747 | 0.659 | 0.732 | 0.805 | 0.793 | 41 |
| **63** | Are the women aware of the importance of attending ANC and PNC consultations? | Maternal health | Background information | MNCAH | 0.744 | 0.829 | 0.744 | 0.720 | 0.683 | 41 |
| **64** | How do we engage adolescents in the design, implementation and monitoring and evaluation of SRH/GBV services in humanitarian settings? | Sexual and reproductive health | Health system strengthening | GBV | 0.744 | 0.707 | 0.720 | 0.768 | 0.780 | 41 |
| **65** | What factors lead to healthy births for women in conflict situations? | Maternal health | Background information | MNCAH | 0.744 | 0.720 | 0.732 | 0.768 | 0.756 | 41 |
| **66** | Which platforms are best suited to deliver health services to adolescents and what promising practices exist that have improved a timely and appropriate response to SRH needs during an acute emergency (preparedness)? | Sexual and reproductive health | Health system strengthening | SRH | 0.738 | 0.720 | 0.720 | 0.756 | 0.756 | 41 |
| **67** | *What are the factors determining health seeking behavior of adolescents in a humanitarian setting? | Adolescent health | Health system strengthening | Crosscutting | 0.735 | 0.775 | 0.706 | 0.735 | 0.725 | 51 |
| **68** | How can high risk pregnancy cases be detected and managed before delivery by trained medical health care providers in humanitarian settings? | Maternal health | Health system strengthening | MNCAH | 0.735 | 0.756 | 0.720 | 0.744 | 0.720 | 41 |
| **69** | What are feasible, acceptable, effective and efficient methods for improving women's experiences of care during pregnancy and childbirth in humanitarian settings? | Maternal health | Health promotion | MNCAH | 0.735 | 0.732 | 0.707 | 0.756 | 0.744 | 41 |
| **70** | What are the caesarean section rates of host displaced people and refugees in different hospitals' serving areas? What are the indications? What are the relative or inappropriate indications that have a bigger impact on this increasing trend? | Maternal health | Background information | MNCAH | 0.732 | 0.780 | 0.756 | 0.707 | 0.683 | 41 |
| **71** | Are ANC visits conducted as proposed in WHO ANC guidelines, especially by means of appropriate context and targeted group? | Maternal health | Background information | MNCAH | 0.732 | 0.732 | 0.756 | 0.720 | 0.720 | 41 |
| **72** | What is the minimal service package that every EMT must have to cover the maternal, child and new born needs during the acute phase of a disaster (Natural or Manmade)? | Maternal health | Health system strengthening | MNCAH | 0.732 | 0.756 | 0.744 | 0.720 | 0.707 | 41 |
| **73** | In humanitarian crisis, what is the effectiveness of child marriage programming on the mental health and psychosocial wellbeing of adolescent girls? | Adolescent health | Health promotion | Mental health | 0.729 | 0.683 | 0.707 | 0.756 | 0.768 | 41 |
| **74** | What interventions are effective to prevent mistreatment and promote dignified care for women during pregnancy, childbirth and postnatal care in humanitarian settings? | Maternal health | Health system strengthening | MNCAH | 0.729 | 0.720 | 0.707 | 0.744 | 0.744 | 41 |
| **75** | What is the frequency and severity, near misses and deaths of WRA with abortion-related complications in humanitarian settings? | Sexual and reproductive health | Background information | Safe abortion | 0.729 | 0.683 | 0.683 | 0.780 | 0.768 | 41 |
| **76** | How do girl-centered interventions equip adolescent girls, their families or guardians, and other authorities to prevent and reduce the risk of gender-based violence, including sexual violence and trafficking? | Sexual and reproductive health | Health promotion | GBV | 0.729 | 0.695 | 0.683 | 0.756 | 0.780 | 41 |
| **77** | What are effective models and examples of FP service delivery in crisis contexts (acute, protracted) in an effective manner to increase coverage and use, and what have we learned from implementation of FP program across diverse contexts? | Sexual and reproductive health | Health system strengthening | Contraception | 0.729 | 0.732 | 0.732 | 0.744 | 0.707 | 41 |
| **78** | What types of community based (culturally sensitive) health education interventions could help adolescents to prevent unwanted pregnancies, STDs and GBV before and during crises? | Sexual and reproductive health | Health promotion | SRH | 0.726 | 0.659 | 0.744 | 0.768 | 0.732 | 41 |
| **79** | What are the promising practices that have ensured a timely and appropriate response to SRH needs during an acute emergency (preparedness)? | Sexual and reproductive health | Health system strengthening | Needs/Challenges | 0.726 | 0.720 | 0.732 | 0.756 | 0.695 | 41 |
| **80** | What is the burden of need/demand for safe abortion care among populations affected by humanitarian crises and what are the key barriers and facilitators to accessing this care? | Sexual and reproductive health | Background information | Safe abortion | 0.726 | 0.695 | 0.707 | 0.744 | 0.756 | 41 |
| **81** | During protracted emergencies, does a community-based awareness program on essential newborn care reduce morbidity and mortality as opposed to standard provision of information at health care clinics? | Newborn health | Health system strengthening | Crosscutting | 0.725 | 0.686 | 0.735 | 0.755 | 0.725 | 51 |
| **82** | In refugee or other displaced populations, does a community volunteer model of home visits to promote home care practices within 48 hours of delivery, accompanied by referral incentives, increase uptake and decrease newborn deaths, as opposed to no community-based intervention? | Newborn health | Health system strengthening | Crosscutting | 0.725 | 0.725 | 0.716 | 0.725 | 0.735 | 51 |
| **83** | Does the integration of group health and nutrition promotion with infant stimulation and play in a safe space within the community, lead to a better wellbeing of the mother and social and cognitive development of children? | Child health | Health promotion | Mental health | 0.723 | 0.735 | 0.686 | 0.725 | 0.745 | 51 |
| **84** | How can respectful maternal health care be effectively delivered in humanitarian setting? | Maternal health | Health system strengthening | MNCAH | 0.723 | 0.720 | 0.720 | 0.732 | 0.720 | 41 |
| **85** | What are the common barriers that hamper patients with STD present their health problems to health facilities/workers? | Sexual and reproductive health | Background information | STD | 0.723 | 0.732 | 0.707 | 0.744 | 0.707 | 41 |
| **86** | Does the provision of organized integrated and inclusive nurturing care for early childhood development through the health services during protracted emergencies promote health and development of children? | Child health | Health promotion | Mental health | 0.721 | 0.716 | 0.667 | 0.755 | 0.745 | 51 |
| **87** | How could we empower adolescents to participate in the development and implementation of health education interventions? | Sexual and reproductive health | Health promotion | SRH | 0.720 | 0.695 | 0.732 | 0.732 | 0.720 | 41 |
| **88** | What brief and targeted interventions can be identified/developed that are feasible and acceptable to improve maternal mental wellbeing in humanitarian settings that can improve women's capacity for providing nurturing care to newborns, infants and young children? | Maternal health | Health promotion | Mental health | 0.720 | 0.720 | 0.707 | 0.720 | 0.732 | 41 |
| **89** | What health sector activities are more needed for pregnant women and children in humanitarian settings? | Maternal health | Health system strengthening | MNCAH | 0.720 | 0.732 | 0.707 | 0.707 | 0.732 | 41 |
| **90** | What is the burden of unsafe abortion for girls and women in a given humanitarian setting of displaced people? | Sexual and reproductive health | Background information | Safe abortion | 0.720 | 0.695 | 0.695 | 0.744 | 0.744 | 41 |
| **91** | *What are the factors determining health seeking behavior of adolescents in a humanitarian setting? | Adolescent health | Health system strengthening | Crosscutting | 0.718 | 0.755 | 0.676 | 0.745 | 0.696 | 51 |
| **92** | Does engagement of adolescent peer groups in health response during an emergency improve their health and social outcomes and mental health? | Adolescent health | Health promotion | Mental health | 0.718 | 0.745 | 0.716 | 0.706 | 0.706 | 51 |
| **93** | Can training health providers on psychological first aid and identification and referral of people with mental illness be an effective public health approach in the absence of trained mental health providers? What are the factors for success? | Sexual and reproductive health | Health system strengthening | Mental health | 0.716 | 0.732 | 0.695 | 0.744 | 0.695 | 41 |
| **94** | What specific actions are being implemented to improve health conditions of pregnant women, lactating women and children during emergencies and what are the impacts of emergencies on their health conditions? | Maternal health | Health promotion | MNCAH | 0.716 | 0.732 | 0.720 | 0.720 | 0.695 | 41 |
| **95** | Does the utilization of local people in the affected area lead to increase the accessibility of blood when needed, if we conduct global promotion or education for them? | Sexual and reproductive health | Health promotion | Crosscutting | 0.716 | 0.707 | 0.720 | 0.732 | 0.707 | 41 |
| **96** | What type of programs are effective at supporting the mental health and SRH for adolescents in emergencies | Sexual and reproductive health | Health system strengthening | Mental health | 0.713 | 0.695 | 0.683 | 0.744 | 0.732 | 41 |
| **97** | Can the assistance of a team of midwives increase the detection of cases of gender violence in adolescent women in a humanitarian emergency situation? | Sexual and reproductive health | Background information | GBV | 0.713 | 0.695 | 0.707 | 0.732 | 0.720 | 41 |
| **98** | For wanted pregnancies, what ANC services are available for women and are these services meeting women’s needs (time, location, accessibility, considering alternative models etc.)? | Maternal health | Background information | MNCAH | 0.713 | 0.732 | 0.720 | 0.732 | 0.671 | 41 |
| **99** | How can SRH health services be most efficiently delivered outside of health and clinic services settings, taking full advantage of new technologies? | Sexual and reproductive health | Health system strengthening | Needs/Challenges | 0.713 | 0.707 | 0.695 | 0.732 | 0.720 | 41 |
| **100** | Does real-time service data availability to program managers and supervisors improve decision-making for newborn health services in humanitarian setting? | Newborn health | Health system strengthening | Crosscutting | 0.713 | 0.725 | 0.706 | 0.735 | 0.686 | 51 |
| **101** | Can the use of a simple diagnostic and treatment algorithm for children with sepsis in humanitarian settings produce the same outcome as a standard management? | Child health | Care of a sick individual | Infectious disease | 0.713 | 0.716 | 0.686 | 0.745 | 0.706 | 51 |
| **102** | Can mobile phones or tablet computers be used to collect and process good quality data on child health for rapid action in humanitarian emergencies | Child health | Health system strengthening | Other | 0.713 | 0.775 | 0.735 | 0.706 | 0.637 | 51 |
| **103** | Does mid upper arm circumference (MUAC) identify newborn infants at high risk of mortality in humanitarian crises? | Newborn health | Background information | Crosscutting | 0.711 | 0.706 | 0.784 | 0.706 | 0.647 | 51 |
| **104** | How does WHO’s current "antenatal care models with a minimum of eight contacts" work in such settings? | Maternal health | Background information | MNCAH | 0.710 | 0.744 | 0.720 | 0.707 | 0.671 | 41 |
| **105** | How can disaster risk management and community preparedness for SRH activities/services improve response to a humanitarian emergency? | Sexual and reproductive health | Health system strengthening | Needs/Challenges | 0.710 | 0.683 | 0.707 | 0.732 | 0.720 | 41 |
| **106** | How do we capture data on number of pregnant women and childbirth in humanitarian settings where most of the deliveries take place at home? | Maternal health | Health system strengthening | MNCAH | 0.710 | 0.695 | 0.659 | 0.732 | 0.756 | 41 |
| **107** | Can specially adapted community case management services (iCCM) improve child health in pastoralist, hard to reach areas in humanitarian settings ? | Child health | Health system strengthening | Infectious disease | 0.708 | 0.725 | 0.667 | 0.725 | 0.716 | 51 |
| **108** | Does systematic screening for sexual exploitation among 10-17 year-old children living in temporary settlements lead to increased prevention, identification and protection of victims, as opposed to not conducting any systematic screening? How should it be planned and implemented? | Sexual and reproductive health | Health system strengthening | GBV | 0.707 | 0.659 | 0.671 | 0.744 | 0.756 | 41 |
| **109** | Is there an affordable hospital service to treat maternal complications in humanitarian emergency settings and low-resources areas? | Maternal health | Background information | MNCAH | 0.707 | 0.707 | 0.732 | 0.720 | 0.671 | 41 |
| **110** | Do the mobile clinic services contribute to reducing maternal mortalities during protracted emergencies? | Maternal health | Health system strengthening | MNCAH | 0.704 | 0.707 | 0.671 | 0.720 | 0.720 | 41 |
| **111** | Can use of modified method of monitoring labour  that involves participation of mothers improve pregnancy outcomes in congested labour wards of health facilities within settlements camps? | Maternal health | Health system strengthening | MNCAH | 0.704 | 0.707 | 0.732 | 0.695 | 0.683 | 41 |
| **112** | What specific models for programming  work best in humanitarian settings to incorporate men's support to women for SRH services? | Sexual and reproductive health | Health system strengthening | Needs/Challenges | 0.704 | 0.683 | 0.695 | 0.732 | 0.707 | 41 |
| **113** | Does the availability of special newborn care increase the survival rate among sick newborns living in temporary settlement during protracted emergencies compared to those area that does not have the special care services | Newborn health | Care of a sick individual | Crosscutting | 0.703 | 0.735 | 0.706 | 0.716 | 0.657 | 51 |
| **114** | To what extent is data collected and analyzed in humanitarian settings to inform programming on SRH issues? Are current tools adequate to include SRH and if not, how can they be improved and developed? Are there systemic problems, for example in the health cluster system that need to be addressed? | Sexual and reproductive health | Health system strengthening | SRH | 0.701 | 0.720 | 0.707 | 0.720 | 0.659 | 41 |
| **115** | What factors contribute to overuse of antibiotics in extreme resource limited settings in protracted emergencies? | Child health | Background information | Other | 0.701 | 0.735 | 0.686 | 0.755 | 0.627 | 51 |
| **116** | What is the effectiveness and cost of different emergency preparedness strategies and plans in reducing neonatal mortality and morbidity compared to no preparedness ? | Newborn health | Health system strengthening | Crosscutting | 0.701 | 0.696 | 0.696 | 0.716 | 0.696 | 51 |
| **117** | What specific barriers do teenage girls experience when trying to access Safe Abortion Care services, as opposed to adult women? | Sexual and reproductive health | Health system strengthening | SRH | 0.698 | 0.671 | 0.659 | 0.744 | 0.720 | 41 |
| **118** | What are the risk factors for and protective factors against adolescents being trafficked during state emergencies? | Sexual and reproductive health | Background information | GBV | 0.698 | 0.671 | 0.659 | 0.756 | 0.707 | 41 |
| **119** | How can pregnant women and those recently given birth (and affected by conflict) be best supported to prevent them developing mental health issues? | Maternal health | Health system strengthening | Mental health | 0.698 | 0.720 | 0.695 | 0.671 | 0.707 | 41 |
| **120** | How can prenatal care for pregnant women be improved to lead to a reduction in perinatal mortality and morbidity in humanitarian settings? | Maternal health | Health system strengthening | MNCAH | 0.698 | 0.671 | 0.646 | 0.744 | 0.732 | 41 |
| **121** | What kind of services should be put in place in order to ensure safer deliveries in humanitarian settings? | Maternal health | Health system strengthening | MNCAH | 0.698 | 0.707 | 0.695 | 0.707 | 0.683 | 41 |
| **122** | In which ways and which settings are the SRH services under-served in emergency situations? | Sexual and reproductive health | Background information | MNCAH | 0.698 | 0.695 | 0.671 | 0.720 | 0.707 | 41 |
| **123** | Does the provision of organized pre-school and school promote cognitive and social development and decrease the risk of PTSD among 3-10 –year old children living in temporary settlements during protracted emergencies, as opposed to no pre-school or school services? | Child health | Health promotion | Mental health | 0.696 | 0.706 | 0.647 | 0.745 | 0.686 | 51 |
| **124** | How can pregnant adolescents be empowered in a humanitarian setting? | Sexual and reproductive health | Health promotion | SRH | 0.695 | 0.671 | 0.659 | 0.732 | 0.720 | 41 |
| **125** | What is needed to address the demand and supply side barriers of uptake of medical and psychosocial services for survivors of Sexual and Gender Based Violence in humanitarian emergencies, in order to create an enabling environment allowing access to comprehensive care and referral to protection, legal and social services for men, boys, women and girls? | Sexual and reproductive health | Health system strengthening | GBV | 0.695 | 0.683 | 0.622 | 0.744 | 0.732 | 41 |
| **126** | What do we know about prenatal care, and birthing conditions for women in conflict situations? | Maternal health | Health system strengthening | MNCAH | 0.695 | 0.707 | 0.707 | 0.671 | 0.695 | 41 |
| **127** | How can clinical management of pregnant women be done effectively during infectious disease outbreaks, such as Ebola, Lassa, Plague? | Maternal health | Health system strengthening | MNCAH | 0.692 | 0.659 | 0.659 | 0.756 | 0.695 | 41 |
| **128** | In the event of a humanitarian emergency, can an inclusive preparedness plan, as opposed to a standard contingency plan, improve the access to primary healthcare for refugee children with disabilities? | Child health | Health system strengthening | Other | 0.691 | 0.706 | 0.647 | 0.706 | 0.706 | 51 |
| **129** | Does the use of community-based adolescent pregnancy surveillance lead to a reduction in newborn mortality? | Newborn health | Health promotion | Crosscutting | 0.691 | 0.667 | 0.637 | 0.745 | 0.716 | 51 |
| **130** | Does sexual and reproductive health education promote uptake of immunization among adolescents? | Sexual and reproductive health | Health promotion | SRH | 0.689 | 0.720 | 0.744 | 0.683 | 0.610 | 41 |
| **131** | What kind of health education programs can be conducted with refugee women to improve their knowledge and attitudes on psychology, social relationships, SRH and child development? | Maternal health | Health promotion | Mental health | 0.689 | 0.695 | 0.707 | 0.671 | 0.683 | 41 |
| **132** | Does provision of basic emergency obstetric care through a (primary ?) health center increase the use of services and coverage for women and reduce excess maternal mortality during humanitarian crises as opposed to basic emergency obstetric care at secondary hospital settings? | Maternal health | Health system strengthening | MNCAH | 0.689 | 0.695 | 0.683 | 0.707 | 0.671 | 41 |
| **133** | What are the main risk factors for tuberculosis transmission among children, living in camps after a natural disaster? | Child health | Background information | Infectious disease | 0.689 | 0.735 | 0.676 | 0.686 | 0.657 | 51 |
| **134** | Does the use of community volunteers lead to increased uptake of contraceptive services among young girls 10-19 in humanitarian emergencies? | Sexual and reproductive health | Health promotion | Contraception | 0.686 | 0.707 | 0.695 | 0.695 | 0.646 | 41 |
| **135** | How can cash transfers effectively increase the access to and use of SRH services in humanitarian settings? How do cash-based interventions influence SRH outcomes in humanitarian settings? | Sexual and reproductive health | Health system strengthening | Needs/Challenges | 0.683 | 0.720 | 0.695 | 0.671 | 0.646 | 41 |
| **136** | What are the drivers of mistreatment in pregnancy and childbirth in humanitarian contexts, across the types of humanitarian settings (acute, protracted, fragile, recovery)? | Maternal health | Background information | MNCAH | 0.683 | 0.683 | 0.646 | 0.707 | 0.695 | 41 |
| **137** | How much reduction of child mortality can be achieved through implementation of ETAT (Emergency triage assessment and treatment) in humanitarian crisis settings? | Child health | Care of a sick individual | Crosscutting | 0.681 | 0.676 | 0.676 | 0.706 | 0.667 | 51 |
| **138** | Can the assistance of a team of midwives increase the detection of cases of gender violence in adolescent women in a humanitarian emergency situation? | Adolescent health | Health promotion | GBV | 0.681 | 0.696 | 0.657 | 0.676 | 0.696 | 51 |
| **139** | What interventions effectively reduce violence against adolescent females and males in humanitarian settings? | Adolescent health | Health promotion | Violence, injuries, trauma | 0.681 | 0.667 | 0.637 | 0.706 | 0.716 | 51 |
| **140** | Does involvement of TBAs in a community increase the probability that women in reproductive age seek more family planning consultations and contraception compared to communities where TBAs do not get involved in promoting it? | Maternal health | Background information | Contraception | 0.680 | 0.695 | 0.695 | 0.683 | 0.646 | 41 |
| **141** | What promising practices exist that have improved the transition from the MISP to comprehensive SRH (Transition MISP to comprehensive)? | Sexual and reproductive health | Health system strengthening | Needs/Challenges | 0.680 | 0.659 | 0.683 | 0.683 | 0.695 | 41 |
| **142** | Does the provision and accessibility of maternal waiting spaces in emergency contexts result in better maternal and neonatal outcomes as opposed to no maternal waiting spaces? | Maternal health, Newborn health | Health system strengthening | MNCAH | 0.677 | 0.671 | 0.683 | 0.695 | 0.659 | 41 |
| **143** | What service delivery mechanisms can be found to be most impactful and cost-effective for the roll out of family planning services within the MISP? | Sexual and reproductive health | Health system strengthening | Needs/Challenges | 0.677 | 0.683 | 0.683 | 0.683 | 0.659 | 41 |
| **144** | What are the physical and mental health impacts of forced/child/early marriage of adolescents living in temporary settlements during a protracted humanitarian emergency? | Adolescent health | Background information | Mental health | 0.677 | 0.683 | 0.622 | 0.683 | 0.720 | 41 |
| **145** | Using a socio-ecological model, which factors are associated with an increased risk of sex and/or labor trafficking among adolescents living in temporary settlements during protracted emergencies? | Sexual and reproductive health | Background information | Occupational health | 0.677 | 0.659 | 0.646 | 0.707 | 0.695 | 41 |
| **146** | How to identify infants 1 month to < 6 months of age that are at highest risk of death in a humanitarian crisis | Child health | Health system strengthening | Crosscutting | 0.676 | 0.608 | 0.608 | 0.765 | 0.725 | 51 |
| **147** | During protracted emergencies, does provision of defined support to home-care for selected high-risk newborns reduce their mortality and hospitalization or improve other health outcomes? | Newborn health | Health promotion | Crosscutting | 0.674 | 0.676 | 0.608 | 0.696 | 0.716 | 51 |
| **148** | What are the different SRH needs across the different types of humanitairan contexts such as refugees, IDPs, acute and protracted conflicts and how are these different needs assessed and accounted in service implementation? | Sexual and reproductive health | Background information | SRH | 0.674 | 0.671 | 0.671 | 0.695 | 0.659 | 41 |
| **149** | Which minimal data set is essential and should always be collected to assess processes and results at single facility level within a RMNCH project in a humanitarian emergency setting? | Maternal health | Background information | MNCAH | 0.674 | 0.683 | 0.683 | 0.695 | 0.634 | 41 |
| **150** | How can we elaborate an indicator measuring the appropriateness of caesarean section rates at facility level in humanitarian emergency settings? | Maternal health | Health system strengthening | MNCAH | 0.674 | 0.671 | 0.671 | 0.707 | 0.646 | 41 |
| **151** | Does the use of lay health workers help adolescents living with HIV to improve adherence and retention in care in humanitarian emergencies, compared to facility/heath worker based interventions? | Sexual and reproductive health | Health system strengthening | HIV | 0.671 | 0.671 | 0.683 | 0.646 | 0.683 | 41 |
| **152** | How does distribution of misoprostol to all pregnant women during the antenatal period for self administration in the third stage of labour impact on maternal mortality from postpartum hemorrhage? Can the community-based distribution help for an increased prevention? | Maternal health | Health promotion | MNCAH | 0.671 | 0.695 | 0.659 | 0.646 | 0.683 | 41 |
| **153** | What kind of mental health assessments and management of disorders among HIV infected adolescents in refugee settlement camps needed to improve their adherence to antiretroviral therapy? | Adolescent health | Health system strengthening | Mental health, HIV | 0.671 | 0.634 | 0.671 | 0.683 | 0.695 | 41 |
| **154** | Can new innovative community case management services (iCCM) improve child health in humanitarian settings ? | Child health | Health system strengthening | Infectious disease | 0.669 | 0.676 | 0.637 | 0.686 | 0.676 | 51 |
| **155** | What are the economic costs of not providing SRH services/access to adolescents? | Sexual and reproductive health | Background information | SRH | 0.668 | 0.646 | 0.622 | 0.707 | 0.695 | 41 |
| **156** | How can SRH programming prevent and mitigate the occurring of GBV, including harmful traditional practices (early and forced-child marriage, female genital mutilation) and unintended pregnancies among adolescent girls from intimate partner violence and conflict related sexual violence? | Sexual and reproductive health | Health system strengthening | GBV | 0.668 | 0.610 | 0.622 | 0.707 | 0.732 | 41 |
| **157** | In a humanitarian emergency, how does psychological trauma impact intention to breastfeed, breastfeeding initiation, duration and self-efficacy among women who are pregnant or who have given birth in the past 12 months? | Maternal health | Background information | MNCAH | 0.668 | 0.683 | 0.634 | 0.695 | 0.659 | 41 |
| **158** | Among families with infants and young children in refuge camp or other temporary settlements, does peer support or or increased support by the health staff promote early child development and mental health, as opposed to standard under-five-clinic follow-up | Child health | Health promotion | Mental health | 0.667 | 0.667 | 0.608 | 0.696 | 0.696 | 51 |
| **159** | Does management of common mental disorders improve SRH outcomes (e.g. contraceptive use, HIV prevalence, delayed sexual activity, reduce teenage pregnancy)? | Sexual and reproductive health | Care of a sick individual | Mental health | 0.665 | 0.659 | 0.646 | 0.683 | 0.671 | 41 |
| **160** | Does the availability and function of temporary maternity waiting homes improve the quality of newborn care in humanitarian situations? | Newborn health | Health system strengthening | Crosscutting | 0.662 | 0.696 | 0.647 | 0.667 | 0.637 | 51 |
| **161** | What are the main risk factors for the poor outcome in refugee children with tuberculosis? | Child health | Background information | Infectious disease | 0.662 | 0.735 | 0.618 | 0.676 | 0.618 | 51 |
| **162** | How can we best measure the implementation of the MISP? | Sexual and reproductive health | Health system strengthening | Needs/Challenges | 0.662 | 0.646 | 0.707 | 0.671 | 0.622 | 41 |
| **163** | During protracted emergencies, does the implementation of evidence-based injury prevention measures, as opposed to no preventive measures, decrease the total number of injuries among children In temporary settlements? | Child health | Health promotion | Violence, injuries, trauma | 0.659 | 0.716 | 0.676 | 0.657 | 0.588 | 51 |
| **164** | Can facilitated youth groups be used to assess and address the psychosocial needs of adolescents after an emergency? | Adolescent health | Health promotion | Mental health | 0.659 | 0.696 | 0.647 | 0.657 | 0.637 | 51 |
| **165** | What is the feasibility and effectiveness of cash-based interventions in enhancing people's access and use of SRH services in humanitarain settings, with special consideration to child/adolescent bride in conflict areas? | Sexual and reproductive health | Health system strengthening | SRH | 0.659 | 0.659 | 0.634 | 0.659 | 0.683 | 41 |
| **166** | What aspects of early child development are most affected in various types of emergencies? | Child health | Background information | Mental health | 0.657 | 0.657 | 0.588 | 0.716 | 0.667 | 51 |
| **167** | Do educational and ECD interventions impact positively children's learning, behaviour, attitudes and beliefs in refugee camps and other marginalized areas? | Child health | Health promotion | Mental health | 0.657 | 0.676 | 0.657 | 0.647 | 0.647 | 51 |
| **168** | What are the real burden and psychological impacts of different types of violence among refugee adolescents? | Adolescent health | Background information | Violence, injuries, trauma | 0.657 | 0.667 | 0.598 | 0.696 | 0.667 | 51 |
| **169** | Can maternal stress during pregnancy be reduced by integrating antenatal care with care pathways tailored to the individual, dealing with problems associated with the social environment of the woman and her family (such as housing, income, nutrition, matters of hygiene etc.)? | Maternal health | Health promotion | Mental health | 0.655 | 0.671 | 0.622 | 0.659 | 0.671 | 41 |
| **170** | How can advanced newborn resuscitation interventions be implemented in refugee camps in low-income settings | Newborn health | Care of a sick individual | Non-communicable disease | 0.654 | 0.735 | 0.637 | 0.627 | 0.618 | 51 |
| **171** | Does a community awareness campaign with education on prevention strategies increase problem awareness and reduce the incidence of childhood drowning? | Child health | Health promotion | Violence, injuries, trauma | 0.654 | 0.716 | 0.657 | 0.637 | 0.608 | 51 |
| **172** | Does the use MWH contribute to improve maternal and neonatal outcomes, as opposed to traveling to the health facility once delivery/complications occur? | Maternal health | Health system strengthening | MNCAH | 0.652 | 0.634 | 0.659 | 0.671 | 0.646 | 41 |
| **173** | What brief and targeted interventions can be developed for men/fathers to increase their engagement/support to the health/mental health needs of women and the nurturing care needs of newborns, infants, and young children? | Child health | Health promotion | Crosscutting | 0.652 | 0.676 | 0.598 | 0.657 | 0.676 | 51 |
| **174** | *What is the stated need for contraception, met and unmet, at various stages of crisis? | Sexual and reproductive health | Background information | Contraception | 0.652 | 0.667 | 0.627 | 0.657 | 0.657 | 51 |
| **175** | Does an early childhood pre-school intervention with holistic psychosocial approach promote wellbeing and development among refugee or asylum seeker children or their parents and support integration into the new home-land? | Child health | Health promotion | Mental health | 0.650 | 0.686 | 0.598 | 0.637 | 0.676 | 51 |
| **176** | What mix-methods are required to improve contraceptive continuation rates among adolescents in humanitarian setting? | Sexual and reproductive health | Health promotion | Contraception | 0.649 | 0.610 | 0.671 | 0.659 | 0.659 | 41 |
| **177** | How do non-access to contraception and non-availability of choices in SRH care affect the quality of life and future plans of women aged 20 and older? Is available obstetric care at temporary settlement adequate? | Sexual and reproductive health | Health system strengthening | Unwanted pregnancy | 0.649 | 0.573 | 0.634 | 0.695 | 0.695 | 41 |
| **178** | Does routine screening for violence (inclusive of Intimate Partner Violence and Sexual Violence) in pregnant women at first antenatal contact with health care providers increase disclosure throughout pregnancy, and therefore improve maternal and neonatal outcomes and access to associated services in humanitarian settings? | Maternal health | Health system strengthening | MNCAH | 0.649 | 0.598 | 0.622 | 0.695 | 0.683 | 41 |
| **179** | How the participatory (adolescent focused, adolescent lead or adolescent centered) process is ensured in SRH and SRMNCAH in humanitarian emergencies? | Sexual and reproductive health | Health system strengthening | SRH | 0.646 | 0.634 | 0.634 | 0.646 | 0.671 | 41 |
| **180** | What are the vulnerabilities experienced by women and children during humanitarian crisis and what protocols can aid organizations put in place to mitigate these conditions? | Maternal health, Newborn health | Health system strengthening | Needs/Challenges | 0.646 | 0.646 | 0.610 | 0.671 | 0.659 | 41 |
| **181** | How does the choice of service delivery platform affect coverage and cure rates for treatment of acute malnutrition in crisis settings? | Child health | Health system strengthening | Undernutrition | 0.645 | 0.637 | 0.618 | 0.676 | 0.647 | 51 |
| **182** | Does the use of community health volunteers reduce neonatal death and late fresh stillbirths in crisis settings? | Newborn health | Health system strengthening | Crosscutting | 0.645 | 0.598 | 0.608 | 0.696 | 0.676 | 51 |
| **183** | What are promising or successful strategies for transitioning a supply chain management system from relying on RH Kits for MISP implementation, to sustainable SRH supply lines for comprehensive SRH service delivery? How do these best practices differ in the recovery phase versus in protracted crises? | Sexual and reproductive health | Health system strengthening | Needs/Challenges | 0.643 | 0.598 | 0.646 | 0.695 | 0.634 | 41 |
| **184** | What are the criteria for starting an antibiotic treatment for Asymptomatic bacteriuria when only urine dipstick is available? Could microscope (fresh sediment without staining) have an additional role of  for these criteria? | Sexual and reproductive health | Care of a sick individual | Crosscutting | 0.643 | 0.683 | 0.634 | 0.646 | 0.610 | 41 |
| **185** | How can effective and cost effective surveillance systems be established for common childhood illnesses among basic school children in a humanitarian setting involving those out of school in the early detection of cases? | Child health | Health system strengthening | Crosscutting | 0.642 | 0.657 | 0.588 | 0.667 | 0.657 | 51 |
| **186** | To what extent does conflict/emergency influence perception towards adoption of particular contraceptive methods among young girls aged 10-19? | Sexual and reproductive health | Background information | Contraception | 0.640 | 0.622 | 0.622 | 0.659 | 0.659 | 41 |
| **187** | Does substantial nutritional and psychological support reduce the incidence of preterm birth in the context of humanitarian emergencies? | Maternal health | Health promotion | MNCAH | 0.640 | 0.622 | 0.622 | 0.671 | 0.646 | 41 |
| **188** | Do the current approaches to SRH in humanitarian emergencies safeguard pregnant woman? | Maternal health | Background information | MNCAH | 0.640 | 0.585 | 0.598 | 0.695 | 0.683 | 41 |
| **189** | How can we give an adequate assistance to the pregnant women at the moment of the delivery in the context of a humanitarian emergency? | Maternal health | Health system strengthening | MNCAH | 0.637 | 0.646 | 0.646 | 0.634 | 0.622 | 41 |
| **190** | What are the healthcare needs and experiences of trafficked women in humanitarian crisis and what can the humanitarian community do to mitigate these harms? | Maternal health | Background information | MNCAH | 0.637 | 0.634 | 0.610 | 0.646 | 0.659 | 41 |
| **191** | What are successful interventions for rapidly establishing SRH supply chains and ensuring SRH commodity security in acute emergency response, outside of pre-positioning supplies? How can we strategically pre-position SRH supplies with differing shelf lives, storage requirements, and demand by setting? What supplies in what quantities, and where? How can we maximize the value for money, or cost effectiveness, of these investments? What strategies could be developed to mitigate wastage (such as coordination systems that move supplies from one place where they are not needed, to another place where they would be used)? | Sexual and reproductive health | Health system strengthening | Needs/Challenges | 0.634 | 0.622 | 0.622 | 0.659 | 0.634 | 41 |
| **192** | How can we protect women from violence and take care of pregnant and newborns, and prevent their violence related diseases and deaths? | Sexual and reproductive health | Health system strengthening | GBV | 0.632 | 0.578 | 0.578 | 0.716 | 0.657 | 51 |
| **193** | How can we protect women from violence and take care of pregnant and newborns, and prevent their violence related diseases and deaths? | Sexual and reproductive health | Health promotion | GBV | 0.631 | 0.598 | 0.622 | 0.659 | 0.646 | 41 |
| **194** | How to provide adequate post-natal care and follow-up during protracted humanitarian emergencies? | Newborn health | Health system strengthening | Crosscutting | 0.627 | 0.598 | 0.588 | 0.667 | 0.657 | 51 |
| **195** | *How to diagnose tuberculosis in in young children in humanitarian crises?* | Child health | Background information | Infectious disease | 0.627 | 0.686 | 0.588 | 0.647 | 0.588 | 51 |
| **196** | Is there a specific model of care that works best for obstetric emergencies in humanitarian settings? What models have proved as most adaptable and effective and what does it cost to provide these services? In addition to access issues, what characterizes technical and interpersonal quality of care in those settings? As the prevalence of NCDs in pregnant women increases, are there lessons to be learned from these models that can inform a broader array of services? | Maternal health | Health system strengthening | MNCAH | 0.622 | 0.634 | 0.598 | 0.634 | 0.622 | 41 |
| **197** | What are the interventions for healthy coping, self-compassion, and psychological adjustment in the aftermath of the traumatic death of a baby/child? | Maternal health | Health promotion | Mental health | 0.619 | 0.634 | 0.610 | 0.634 | 0.598 | 41 |
| **198** | What are the effective approaches to conduct implementation researches (in general and in particular for SRMNCAH) in humanitarian settings, and why are they needed (e.g. identifying the contextual factors)? | Sexual and reproductive health | Health system strengthening | Needs/Challenges | 0.619 | 0.634 | 0.610 | 0.634 | 0.598 | 41 |
| **199** | What factors influence the decision on which services to be provided? | Sexual and reproductive health | Background information | SRH | 0.616 | 0.634 | 0.598 | 0.634 | 0.598 | 41 |
| **200** | Does investing on women's mental health, agency, and physical health through a women-centered approach during and after pregnancy improve health (physical and mental) outcomes for her and her newborn/infant? | Maternal health | Health system strengthening | MNCAH | 0.616 | 0.610 | 0.598 | 0.646 | 0.610 | 41 |
| **201** | *Is it more acceptable for women and adolescents to access GBV services provided at the primary level care through reproductive health services within a community services model versus setting up GBV specific service points? | Adolescent health | Health promotion | GBV | 0.615 | 0.627 | 0.627 | 0.618 | 0.588 | 51 |
| **202** | What are the added risks of babies born due to rape which occurred as a result of the humanitarian crisis? | Newborn health | Background information | MNCAH | 0.613 | 0.585 | 0.585 | 0.622 | 0.659 | 41 |
| **203** | Among newborns in humanitarian settings, does the routine use of bilirubinometers rather than visual inspection lead to more accurate identification of neonatal jaundice and improved neonatal health outcomes? | Newborn health | Background information | Non-communicable disease | 0.613 | 0.667 | 0.647 | 0.578 | 0.559 | 51 |
| **204** | How can community volunteers help to reduce suicide among adolescents in a humanitarian crisis? | Adolescent health | Health system strengthening | Violence, injuries, trauma | 0.613 | 0.627 | 0.598 | 0.618 | 0.608 | 51 |
| **205** | *Can community-based care be a safe and feasible option of clinical care for survivors of sexual violence in a setting where insecurity, and other challenges act as barriers to facility-based care, and what are the challenges to providing care in this manner? | Sexual and reproductive health | Care of a sick individual | GBV | 0.613 | 0.569 | 0.549 | 0.667 | 0.667 | 51 |
| **206** | Can simplified verbal autopsy tools be adapted for use in emergency settings to capture the main causes of neonatal mortality? | Newborn health | Health system strengthening | Crosscutting | 0.610 | 0.627 | 0.569 | 0.647 | 0.598 | 51 |
| **207** | How can quality maternal care (ANC, delivery, PNC) be provided to  prevent morbidity and mortality among pregnant/postpartum women living in the protected settlements who are vulnerable condition compared to the women living in a safer environment? | Maternal health | Health system strengthening | MNCAH | 0.607 | 0.585 | 0.598 | 0.610 | 0.634 | 41 |
| **208** | In humanitarian settings, can male-targeted interventions promote comprehensive nurturing care among newborns, infants and young children with father as the primary caregiver? | Child health | Health promotion | Mental health | 0.605 | 0.627 | 0.578 | 0.618 | 0.598 | 51 |
| **209** | Does mass deployment of educational courses for safe deliveries and neonatal resuscitation decrease the incidence of unsafe births? | Newborn health | Health promotion | Crosscutting | 0.605 | 0.588 | 0.549 | 0.686 | 0.598 | 51 |
| **210** | How do the delivery strategies of health service in humanitarian contexts differ based on the strength/robustness/preparedness of underlying health systems’ (other systems)? | Maternal health | Health system strengthening | Needs/Challenges | 0.604 | 0.585 | 0.598 | 0.634 | 0.598 | 41 |
| **211** | What interventions are needed to improve health seeking behaviour of adolescents/youth age group and prevent substance use? | Adolescent health | Health promotion | Mental health | 0.603 | 0.588 | 0.578 | 0.637 | 0.608 | 51 |
| **212** | What do positive deviants (i.e., girls and their families who have been protected from sexual and gender based violence) do differently than those who are subject to SGBV that can help to design better ways to protect girls in temporary settlements? | Sexual and reproductive health | Background information | GBV | 0.598 | 0.549 | 0.549 | 0.622 | 0.671 | 41 |
| **213** | *Does the current programming in emergencies meet the contraceptive needs and ensure continuous supply without stock outs for women who have given birth in the last 12 months? | Sexual and reproductive health | Background information | Contraception | 0.596 | 0.618 | 0.598 | 0.598 | 0.569 | 51 |
| **214** | Is there an added value of a < 6 weeks (pregnancy) consultation to address unwanted pregnancy in humanitarian emergencies? | Sexual and reproductive health | Health system strengthening | Unwanted pregnancy | 0.595 | 0.573 | 0.537 | 0.646 | 0.622 | 41 |
| **215** | How to identify factors leading to a responsive health system that can manage delivery of quality 24/7 health services to high risk pregnant/ postpartum women and ensure good health of their fetus or newborns (28 days) in an emergency setting versus to delivery of routine health care services? | Maternal health | Health system strengthening | MNCAH | 0.595 | 0.573 | 0.585 | 0.622 | 0.598 | 41 |
| **216** | In high stress sites, can minimally trained or untrained personnel equipped with essential packages of medications, support materials, and diagnostics provide as good newborn care as personnel familiar with newborn care? | Newborn health | Health system strengthening | Crosscutting | 0.581 | 0.549 | 0.569 | 0.627 | 0.578 | 51 |
| **217** | Does the provision of prefilled, single-dose injection devices which combine medication, syringe, and needle in a small, sterile package (E.g. Uniject®) improve neonatal outcomes following | Newborn health | Care of a sick individual | Infectious disease | 0.581 | 0.618 | 0.559 | 0.608 | 0.539 | 51 |
| **218** | In a humanitarian emergency, what steps can the State take to keep 1 month to 9 year old children healthy and properly nourished? | Child health | Health system strengthening | Crosscutting | 0.581 | 0.529 | 0.490 | 0.676 | 0.627 | 51 |
| **219** | What interventions promote resilience in early brain development? | Child health | Background information | Mental health | 0.578 | 0.588 | 0.510 | 0.618 | 0.598 | 51 |
| **220** | How have suffering of recent conflict, recurring natural disasters, or both devastated adolescents knowledge on self and body protection, sexual maturity? | Sexual and reproductive health | Background information | SRH | 0.573 | 0.573 | 0.549 | 0.573 | 0.598 | 41 |
| **221** | What are the target product profiles of the top 3-5 demanded SRHR products and services? | Sexual and reproductive health | Health system strengthening | Needs/Challenges | 0.564 | 0.585 | 0.573 | 0.573 | 0.524 | 41 |
| **222** | What is the prevalence of Post-Traumatic Stress Disorders among Forcibly Displaced Myanmar National women who are pregnant or have given birth in the last 12 months and who are living in Cox Bazar, Bangladesh? | Maternal health | Background information | Mental health | 0.558 | 0.622 | 0.573 | 0.512 | 0.524 | 41 |
| **223** | Given the nature of humanitarian emergency settings, how much of a priority do RMNCAH issues have for women compared to such issues survival, shelter, security and food? | Maternal health | Background information | MNCAH | 0.555 | 0.585 | 0.537 | 0.549 | 0.549 | 41 |
| **224** | In humanitarian emergencies in middle- and low-income countries, does newborn care by specialised neonatal nurses as opposed to general nurses and midwifes lead to reduced incidence of neonatal infection, respiratory failure, feeding problems or death? | Newborn health | Health system strengthening | Crosscutting | 0.554 | 0.627 | 0.480 | 0.578 | 0.529 | 51 |
| **225** | How can Syrian refugee women achieve the necessary information to protect their children and teach them how to protect themselves in humanitarian emergencies? | Sexual and reproductive health | Health promotion | Crosscutting | 0.552 | 0.537 | 0.549 | 0.561 | 0.561 | 41 |
| **226** | Is there an ethically acceptable upper limit in the intensity of neonatal care in humanitarian emergency settings, different from that in stable conditions and dependent on the availability of personnel and material resources? | Newborn health | Care of a sick individual | Needs/Challenges | 0.549 | 0.539 | 0.480 | 0.608 | 0.569 | 51 |
| **227** | In the context of humanitarian emergencies in LMIC, which approach results in better outcomes for mood, anxiety, and trauma-related disorders among children : Integration of services in medical response or parallel service provision by other specially-grained personnel? | Child health | Care of a sick individual | Mental health | 0.549 | 0.598 | 0.520 | 0.559 | 0.520 | 51 |
| **228** | Among adolescents in a humanitarian crisis context, what are the determinants of psychological resilience, and how can we improve resilience? | Adolescent health | Background information | Mental health | 0.549 | 0.559 | 0.480 | 0.608 | 0.549 | 51 |
| **229** | What are the long term impacts of emergent situations and economic collapses on adolescents' mental health and well-being? | Adolescent health | Background information | Mental health | 0.549 | 0.539 | 0.480 | 0.598 | 0.578 | 51 |
| **230** | *In humanitarian settings, are the women who are pregnant or who recently delivered (i.e. in the last 12 months) exposed to different levels of violence/abuse than married women of the same age group? Is there a difference when compared to the women's home situation? | Sexual and reproductive health | Background information | GBV | 0.549 | 0.578 | 0.500 | 0.559 | 0.559 | 51 |
| **231** | What interventions are needed for adolescents in Nepal to improve the use of FP planning services (especially to prevent unsafe abortions) during the humanitarian emergencies in addition to the existing free abortion services? | Sexual and reproductive health | Health system strengthening | SRH | 0.543 | 0.549 | 0.549 | 0.537 | 0.537 | 41 |
| **232** | How is women's access to sexual and reproductive health care in a specific context, i.e. Afghanistan? | Maternal health | Background information | MNCAH | 0.543 | 0.524 | 0.524 | 0.573 | 0.549 | 41 |
| **233** | Do financial incentives, frequency of supervision, or other types of motivation used for community health workers before a crisis affect how community service provision is sustained during and after the crisis? | Child health | Health system strengthening | Infectious disease | 0.537 | 0.480 | 0.500 | 0.618 | 0.549 | 51 |
| **234** | In humanitarian settings, can childhood mortality be reduced by the provision of further evidence-based recommendations and guidance on medical intensive care? | Child health | Health system strengthening | Crosscutting | 0.537 | 0.588 | 0.490 | 0.539 | 0.529 | 51 |
| **235** | What interventions most reduce the risk of substance abuse, family disharmony, interpersonal violence, and attachment problems in the aftermath of the traumatic death of a baby/child? | Maternal health | Health promotion | Mental health | 0.534 | 0.549 | 0.512 | 0.537 | 0.537 | 41 |
| **236** | How can the basic humanitarian assistance be integrated into disaster fatalities especially in devastated and limited health infrastructure without professional health experts in low or middle income countries? | Adolescent health | Health system strengthening | Needs/Challenges | 0.532 | 0.500 | 0.471 | 0.588 | 0.569 | 51 |
| **237** | In conflicts or other humanitarian emergencies, does provision of donated, safe breast milk lead to improved health outcomes among preterm babies? | Newborn health | Health promotion | Crosscutting | 0.529 | 0.608 | 0.431 | 0.539 | 0.539 | 51 |
| **238** | How can local decision making and priority setting (in the intensity of neonatal and prematurity care) be supported by an ethical approval? | Maternal health | Health system strengthening | MNCAH | 0.527 | 0.500 | 0.524 | 0.549 | 0.537 | 41 |
| **239** | *How can a minimal package of multi-sectoral interventions be determined and established to response to SGBV in emergency settings effectively? | Sexual and reproductive health | Health promotion | GBV | 0.527 | 0.490 | 0.500 | 0.569 | 0.549 | 51 |
| **240** | What interventions for adolescents are needed at the time of humanitarian disasters to mitigate intermediate term outcomes? | Adolescent health | Health promotion | Needs/Challenges | 0.527 | 0.529 | 0.500 | 0.539 | 0.539 | 51 |
| **241** | What innovative implementation and support approaches are associated with sustained and effective practice change of health workers compared to current approaches? | Newborn health | Health system strengthening | Crosscutting | 0.522 | 0.520 | 0.490 | 0.569 | 0.510 | 51 |
| **242** | *In humanitarian emergencies, does the use of community groups and youth clubs along a young voice concept, rather than other interventions implemented by government and other NGOs, reduce maternal and child mortality as opposed to | Adolescent health | Health system strengthening | Crosscutting | 0.520 | 0.520 | 0.510 | 0.520 | 0.529 | 51 |
| **243** | Is there an upper limit in the intensity of neonatal and prematurity care and is it ethically acceptable, necessary and mandatory in humanitarian emergency settings and low-resources areas? | Newborn health | Background information | MNCAH | 0.518 | 0.488 | 0.537 | 0.524 | 0.524 | 41 |
| **244** | How are these similar to/different from the drivers and interventions documented in developing countries? | Maternal health | Health system strengthening | MNCAH | 0.515 | 0.573 | 0.537 | 0.488 | 0.463 | 41 |
| **245** | How can hybrid studies (designed for effectiveness and implementation) better inform about delivering mental health-related services to women and families in LMICS affected by conflict? (What works? How? When?) | Sexual and reproductive health | Background information | Mental health | 0.515 | 0.520 | 0.500 | 0.539 | 0.500 | 51 |
| **246** | Does a technology supported psychological intervention for adolescents experiencing emotional and behavioral problems and functional impairment, reduce problems and increase functioning compared to a control condition? | Adolescent health | Care of a sick individual | Mental health | 0.512 | 0.569 | 0.490 | 0.500 | 0.490 | 51 |
| **247** | How can pelvic floor dysfunctions be diagnosed and managed in emergency situations? | Sexual and reproductive health | Care of a sick individual | Crosscutting | 0.512 | 0.549 | 0.549 | 0.488 | 0.463 | 41 |
| **248** | Can improvements in pharmacological formulations, dosing or presentations improve the outcome of neonatal infections, perinatal asphyxia or the major morbidities related to preterm birth | Newborn health | Care of a sick individual | Crosscutting | 0.510 | 0.529 | 0.500 | 0.500 | 0.510 | 51 |
| **249** | Can the experience of positive deviants (those with a nutritionally good diet) be used to improve children's dietary diversity in humanitarian settings? | Child health | Health promotion | Undernutrition | 0.510 | 0.569 | 0.471 | 0.510 | 0.490 | 51 |
| **250** | Among infants and young children in refugee camps or other temporary settlements, does periodic provision of antibiotics improve post-neonatal under-five mortality? | Child health | Health promotion | Infectious disease | 0.500 | 0.510 | 0.490 | 0.520 | 0.480 | 51 |
| **251** | What is the impact of physical chastisement on mental health outcomes of adolescents (such as depression, trauma and suicide) during humanitarian emergencies? | Adolescent health | Background information | Violence, injuries, trauma | 0.500 | 0.480 | 0.480 | 0.539 | 0.500 | 51 |
| **252** | What are International Organizations and NGO's attitudes and practices for the vulnerability of adolescents in terms of mental health in crisis situations? | Adolescent health | Background information | Mental health | 0.498 | 0.549 | 0.500 | 0.500 | 0.441 | 51 |
| **253** | Are the victims of IPV who are dislocated as result of a natural disasters able to access services and relocation procedures that are appropriate to their needs and to the effects of dislocation in their life? | Sexual and reproductive health | Background information | GBV | 0.497 | 0.488 | 0.476 | 0.512 | 0.512 | 41 |
| **254** | How can one take advantage of all opportunities to promote the development of children in humanitarian emergencies? | Child health | Health system strengthening | Mental health | 0.495 | 0.441 | 0.412 | 0.569 | 0.559 | 51 |
| **255** | *Does use of soccer as a means of information sharing and capacity building improve the access on RH information and delivery among adolescents? | Sexual and reproductive health | Health promotion | Needs/Challenges | 0.495 | 0.539 | 0.500 | 0.480 | 0.461 | 51 |
| **256** | Are Fertility Awareness Based Methods an acceptable form of contraception for women living in temporary settlements during protracted emergencies? Are these methods also acceptable for Forcibly Displaced Myanmar National women living in Cox Bazar, Bangladesh who are of reproductive age? | Sexual and reproductive health | Health system strengthening | Contraception | 0.491 | 0.561 | 0.500 | 0.439 | 0.463 | 41 |
| **257** | Does group antenatal care improve the experience of pregnant women who are Forcibly Displaced Myanmar Nationals living in Cox Bazar, Bangladesh? | Maternal health | Health system strengthening | MNCAH | 0.491 | 0.512 | 0.512 | 0.476 | 0.463 | 41 |
| **258** | *Are the protocols and procedures adequate and designed to attend the needs of sub-populations such as indigenous women, immigrant women and transgender women? | Sexual and reproductive health | Background information | Needs/Challenges | 0.490 | 0.510 | 0.451 | 0.480 | 0.520 | 51 |
| **259** | *Are modern methods of family planning safe enough to be promoted after medical abortion in refugee setting? Can LARCs be promoted after a medical abortion in humanitarian emergencies? | Sexual and reproductive health | Background information | Contraception | 0.490 | 0.520 | 0.480 | 0.490 | 0.471 | 51 |
| **260** | *Can the detection of GBV cases by SRH staff, bring to an early disclosure and treatment of GBV and consequently to a reduction of SRH diseases related/determined by the exposure to GBV? | Adolescent health | Health promotion | GBV | 0.490 | 0.500 | 0.480 | 0.500 | 0.480 | 51 |
| **261** | What is the incidence and risk factors for pelvic floor disorders in emergency situations? | Sexual and reproductive health | Background information | Crosscutting | 0.488 | 0.512 | 0.500 | 0.476 | 0.463 | 41 |
| **262** | How could migrant worker best be used to promote adolescent heath in humanitarian crisin conditions? | Adolescent health | Health system strengthening | Crosscutting | 0.488 | 0.500 | 0.480 | 0.480 | 0.490 | 51 |
| **264** | In humanitarian emergencies, what interventions are effective for promoting adolescent mental health and well-being? | Adolescent health | Health promotion | Mental health | 0.473 | 0.471 | 0.441 | 0.500 | 0.480 | 51 |
| **265** | Are the victims of IPV who are dislocated as result of a natural disasters able to access services and relocation procedures that are appropriate to their needs and to the effects of dislocation in their life? | Adolescent health | Background information | GBV | 0.471 | 0.451 | 0.461 | 0.500 | 0.471 | 51 |
| **266** | *Does use of community groups and youth clubs through young voice concept reduces maternal and child mortality, compared to other interventions implemented by government and other NGOs? | Maternal health, Newborn health | Health system strengthening | Crosscutting | 0.471 | 0.480 | 0.461 | 0.490 | 0.451 | 51 |
| **267** | How can we improve psychological resilience? | Adolescent health | Health promotion | Mental health | 0.468 | 0.431 | 0.412 | 0.529 | 0.500 | 51 |
| **268** | Does increased access to business through leading and borrowing groups empower women to reduce maternal and child mortality | Maternal health | Health promotion | MNCAH | 0.463 | 0.463 | 0.439 | 0.488 | 0.463 | 41 |
| **269** | In humanitarian emergency settings, does routine dengue vaccination with currently available products reduce the incidence of dengue fever or vaccine-related complications among 2-to-5-year-old children? | Child health | Health promotion | Infectious disease | 0.456 | 0.451 | 0.422 | 0.490 | 0.461 | 51 |
| **270** | *In humanitarian emergencies, does the use of local social capital through men's, women's and youth groups generate more effective strategies to reduce maternal and child mortality than the use of general information and knowledge sharing done by government and other NGOs | Maternal health, Newborn health | Health system strengthening | Crosscutting | 0.456 | 0.461 | 0.461 | 0.461 | 0.441 | 51 |
| **271** | Does promoting production and use of traditional foods and herbs like molinga improve nutrition status of pregnant women and lactating mothers | Maternal health | Health promotion | MNCAH | 0.448 | 0.439 | 0.451 | 0.451 | 0.451 | 41 |
| **273** | Does the prevention of urinary and reproductive infections in temporary settlements lead to prevent renal disease among pregnant women and related children deaths? Are the hemodialysis services included in humanitarian emergency teams? | Maternal health | Health system strengthening | MNCAH | 0.418 | 0.451 | 0.402 | 0.415 | 0.402 | 41 |
| **274** | In emergency settings, what is the additive value of neuroprotective strategies in infants with perinatal asphyxia | Newborn health | Health promotion | Non-communicable disease | 0.400 | 0.392 | 0.333 | 0.461 | 0.412 | 51 |
| **275** | Which results in better outcomes for mood, anxiety, and trauma-related disorders in context of humanitarian emergencies in LMIC: Integration of mhGAP-HIG (Humanitarian Intervention Guide) when included/well-integrated with medical response; OR mhGAP-HIG provided by specialty-trained personel but separate to medical care? | Adolescent health | Health promotion | Mental health | 0.358 | 0.382 | 0.373 | 0.353 | 0.324 | 51 |
| **276** | What are the specific criteria for organizing awareness and cognitive awareness in pre-school children and strengthening social communication with their peers in places where | Child health | Health system strengthening | Mental health | 0.331 | 0.343 | 0.343 | 0.324 | 0.314 | 51 |
| **277** | What are the factors associated with stillbirths at Jigme Dorji Wangchuck National Referral hospital in Thimphu? | Maternal health | Background information | MNCAH | 0.320 | 0.366 | 0.341 | 0.293 | 0.280 | 41 |
| **278** | What is the impact of the "XYZ" program on mother's mental health, infants' physical, mental, social (attachment) and cognitive development and children's long-term physical, mental, social and cognitive development? | Child health | Health promotion | Mental health | 0.279 | 0.284 | 0.245 | 0.324 | 0.265 | 51 |
| **279** | *Do new protocols and services for dislocating survivors of IPV need to be created specially for industrialized countries such USA? | Adolescent health | Health promotion | GBV | 0.277 | 0.275 | 0.275 | 0.275 | 0.284 | 51 |
| **280** | How does the adolescent are mainstreamed into psychosocial wellbeing in a humanitarian setting with limited or even zero information on SRH? | Adolescent health | Health promotion | Mental health | 0.262 | 0.284 | 0.255 | 0.265 | 0.245 | 51 |

**Supplementary Table C. Distribution of RPS scores per dimension for the top TEN CHNRI research priority questions per SRMNCAH domain**

***Sexual and reproductive health***

| **Ranking** | **Question** | **Population** | **Approach** | **Addressed Condition** | **Avg RPS** | **Answerable** | **Program**  **feasible** | **Public health relevant** | **Equity value** |
| --- | --- | --- | --- | --- | --- | --- | --- | --- | --- |
| 4 | What are the barriers for adolescents' contraceptive use in humanitarian settings? | Adolescents  (10-19 y) | Background information | Contraception | 0.835 | 0.841 | 0.805 | 0.854 | 0.841 |
| 5 | What are barriers to contraceptive use in humanitarian settings and successful strategies to overcome them? | Women | Health system strengthening | Contraception | 0.835 | 0.817 | 0.829 | 0.854 | 0.841 |
| 6 | Does the community-based distribution of contraceptives to sexually active adolescents contribute to increased prevention of unwanted pregnancies in humanitarian settings, as opposed to the standard provision of contraceptives in the health clinics? | Adolescents  (10-19 y) | Health system strengthening | SRH | 0.829 | 0.829 | 0.817 | 0.841 | 0.829 |
| 8 | Does the provision of adolescent-friendly family planning services in protracted humanitarian emergencies improve their use by 10-19 -year old adolescents, as opposed to standard provision of family planning services? | Adolescents  (10-19 y) | Health system strengthening | SRH | 0.820 | 0.805 | 0.805 | 0.854 | 0.817 |
| 9 | Is community-based distribution of family planning services, including injectables, applicable and feasible in humanitarian settings and does it enhance people’s access to and use of contraceptives? | Women | Health system strengthening | Contraception | 0.817 | 0.866 | 0.805 | 0.817 | 0.780 |
| 10 | Does economic and knowledge empowerment of adolescent girls and young women reduce the prevalence of teenage pregnancies in refugee settlement camps? | Adolescents  (10-19 y) | Health promotion | SRH | 0.814 | 0.768 | 0.805 | 0.866 | 0.817 |
| 18 | What barriers do unaccompanied adolescents face in accessing to SRHR information and services as compared to those accompanied by others? | Adolescents  (10-19 y) | Background information | SRH | 0.799 | 0.793 | 0.793 | 0.793 | 0.817 |
| 23 | What are effective strategies to deliver proven SRH interventions to adolescents in crises settings? | Adolescents  (10-19 y) | Health system strengthening | SRH | 0.790 | 0.768 | 0.793 | 0.805 | 0.793 |
| 24 | What are successful strategies to deliver a full range of contraceptives including long acting methods of contraception from the onset of a humanitarian emergency? | Women | Health system strengthening | Contraception | 0.790 | 0.744 | 0.805 | 0.817 | 0.793 |
| 35 | What are the needs/available resources/attitudes of girls and women to manage menstruation during complex humanitarian emergencies? | Adolescents  (10-19 y) | Background information | SRH | 0.771 | 0.793 | 0.780 | 0.744 | 0.768 |

***Maternal health***

| **Ranking** | **Question** | **Population** | **Approach** | **Addressed Condition** | **Avg RPS** | **Answerable** | **Program feasible** | **Public health relevant** | **Equity value** |
| --- | --- | --- | --- | --- | --- | --- | --- | --- | --- |
| 11 | Does having trained CHWs improve adherence to ANC/PNC and assisted deliveries within displaced populations and to provide better infant care (e.g. exclusive breast feeding, infant stimulation etc.)? | Women | Health system strengthening | MNCAH | 0.814 | 0.805 | 0.805 | 0.829 | 0.817 |
| 15 | Does the provision of training to health workers on WHO recommendations for women-centered care for antenatal care, childbirth and post-natal care improve women's satisfaction with health services, childbirth outcomes (including types and nature of interventions during childbirth, outcomes for newborns) and post-natal health (including maternal morbidity and mental health) | Women | Health system strengthening | MNCAH | 0.805 | 0.841 | 0.817 | 0.793 | 0.768 |
| 16 | What are effective and cost-effective strategies for maintaining and strengthening capacity of skilled birth attendants to detect and manage obstetric and newborn complications in humanitarian settings? | Women | Health system strengthening | MNCAH | 0.805 | 0.793 | 0.780 | 0.841 | 0.805 |
| 17 | In an acute phase emergency, which surveillance methodology is most efficient and effective to capture maternal and perinatal mortality at the community level? | Women | Background information | MNCAH | 0.802 | 0.793 | 0.744 | 0.854 | 0.817 |
| 21 | What are the factors that constrain the utilization of reproductive maternal child and adolescent health (RMNCAH) services by pregnant women in a humanitarian setting? | Women | Health system strengthening | MNCAH | 0.793 | 0.793 | 0.793 | 0.817 | 0.768 |
| 27 | Is the provision of home visits by a skilled attendant to pregnant and postpartum women living in temporary settlements during emergencies more effective, safer and less costly than the use of available standard health facilities? | Women | Health system strengthening | MNCAH | 0.784 | 0.793 | 0.780 | 0.793 | 0.768 |
| 28 | Do the mobile clinics in protracted emergencies lead to adequate antenatal care coverage among displaced people? | Women | Health system strengthening | MNCAH | 0.780 | 0.805 | 0.768 | 0.780 | 0.768 |
| 31 | How can maternal mental health care be integrated into pre-conceptional, antenatal, delivery, and post-partum care in humanitarian settings? What are acceptable, effective and cost-effective strategies? | Women | Health system strengthening | Mental health | 0.774 | 0.768 | 0.744 | 0.793 | 0.793 |
| 32 | Does the provision of women's health services, specifically maternal health services, by skilled providers from the population of displaced persons serve as an effective care model? | Women | Health system strengthening | MNCAH | 0.774 | 0.768 | 0.744 | 0.780 | 0.805 |
| 33 | What tools are available to assist women experiencing pregnancy loss (inclusive of miscarriage, still birth and neonatal death) in humanitarian settings? | Women | Health system strengthening | MNCAH | 0.774 | 0.793 | 0.768 | 0.768 | 0.768 |

***Newborn health***

| **Ranking** | **Question** | **Population** | **Approach** | **Addressed Condition** | **AVG RPS** | **Answerable** | **Program feasible** | **Public health relevant** | **Equity value** |
| --- | --- | --- | --- | --- | --- | --- | --- | --- | --- |
| 1 | What are the common causes of newborn mortality and morbidity in different types of humanitarian emergency? | Newborns | Background information | Crosscutting | 0.873 | 0.902 | 0.833 | 0.902 | 0.853 |
| 2 | Does the implementation of a home-based hygiene promotion program lead to a reduction of neonatal infections? | Newborns | Health promotion | Infectious disease | 0.846 | 0.833 | 0.804 | 0.882 | 0.863 |
| 3 | What are the main causes and underlying determinants of perinatal death in varying humanitarian contexts? | Newborns | Background information | Crosscutting | 0.841 | 0.824 | 0.794 | 0.912 | 0.833 |
| 7 | In emergencies, can Kangaroo mother care (KMC) provide as good health outcomes for preterm babies as incubator care | Newborns | Health system strengthening | Crosscutting | 0.824 | 0.843 | 0.843 | 0.833 | 0.775 |
| 12 | Does a bundle of critical aseptic practices adopted by healthcare providers and caregivers around the time of birth and at home during postnatal care, lead to lower incidence of infections and infection-related neonatal mortality, as compared to, usual care ? | Newborns | Health promotion | Infectious disease | 0.814 | 0.794 | 0.784 | 0.853 | 0.824 |
| 13 | In settlement camps, does counseling of parents about home care for newborns and identification of danger signs before discharge reduce early neonatal mortality? | Newborns | Health promotion | Crosscutting | 0.814 | 0.814 | 0.833 | 0.824 | 0.784 |
| 14 | For babies born in health centres at low-income country refugee camps, is newborn mortality and morbidity less frequent if the attending midwives/TBAs have been trained in specific delivery room procedures than if the health personnel have not been trained in these skills? | Newborns | Health promotion | Crosscutting | 0.809 | 0.824 | 0.824 | 0.814 | 0.775 |
| 19 | Among women in humanitarian crisis contexts, can participation in peer-group sessions during pregnancy increase knowledge about newborn care and improve newborn health outcomes. | Newborns | Health promotion | Crosscutting | 0.797 | 0.794 | 0.765 | 0.853 | 0.775 |
| 25 | Can mobile health services for providing post-natal care to newborns and mothers after a natural disaster decrease mortality and morbidity? | Newborns | Health system strengthening | Infectious disease | 0.789 | 0.814 | 0.765 | 0.804 | 0.775 |
| 29 | Can existing lifesaving interventions to care for small and sick newborns be adapted to be applicable and practical in a humanitarian context? | Newborns | Care of a sick individual | Crosscutting | 0.779 | 0.784 | 0.735 | 0.804 | 0.794 |

***Child health***

| **Ranking** | **Question** | **Population** | **Approach** | **Addressed Condition** | **AVG RPS** | **Answerable** | **Program feasible** | **Public health relevant** | **Equity value** |
| --- | --- | --- | --- | --- | --- | --- | --- | --- | --- |
| 20 | Does identification and management of nutritionally at-risk infants aged <6month reduce morbidity and improve infant growth/development in emergency settings? | Infants or children | Health promotion | Crosscutting | 0.794 | 0.833 | 0.735 | 0.824 | 0.784 |
| 22 | Is community-based management an effective approach for reducing morbidity and mortality from severe acute malnutrition among under five-year-old children humanitarian settings? | Infants or children | Care of a sick individual | Undernutrition | 0.792 | 0.814 | 0.784 | 0.794 | 0.775 |
| 26 | In refugee camps and other temporary settlements, does a targeted hygiene awareness campaign, including provision of self-hygiene materials (soap, information sheets) reduce the incidence of fecal-oral transmitted diseases among children? | Infants or children | Health promotion | Infectious disease | 0.789 | 0.814 | 0.804 | 0.784 | 0.755 |
| 41 | In different kinds of humanitarian settings, can the use of community health workers improve the quality, continuity, and user access to child health services, and reduce costs? | Infants or children | Health system strengthening | Infectious disease | 0.767 | 0.765 | 0.735 | 0.775 | 0.794 |
| 45 | How do current nutrition interventions delivered in refugee camps meet the needs of high-risk infants/children, such as those born preterm, low birth weight, or with perinatal injury? | Infants or children | Health system strengthening | Undernutrition | 0.765 | 0.775 | 0.735 | 0.784 | 0.765 |
| 50 | What are the demographic, social and other factors that are associated with complete childhood vaccination status in conflict-affected countries? | Infants or children | Health promotion | Infectious disease | 0.757 | 0.775 | 0.686 | 0.804 | 0.765 |
| 60 | Does routine oral cholera vaccination in humanitarian emergencies decrease the burden of acute watery diarrhea in children? | Infants or children | Health promotion | Infectious disease | 0.748 | 0.784 | 0.735 | 0.745 | 0.725 |
| 61 | Do community health workers continue to provide curative services during acute crises? | Infants or children | Health system strengthening | Infectious disease | 0.748 | 0.755 | 0.716 | 0.775 | 0.745 |
| 83 | Does the integration of group health and nutrition promotion with infant stimulation and play in a safe space within the community, lead to a better wellbeing of the mother and social and cognitive development of children? | Infants or children | Health promotion | Mental health | 0.723 | 0.735 | 0.686 | 0.725 | 0.745 |
| 86 | Does the provision of organized integrated and inclusive nurturing care for early childhood development through the health services during protracted emergencies promote health and development of children? | Infants or children | Health promotion | Mental health | 0.721 | 0.716 | 0.667 | 0.755 | 0.745 |

***Adolescent health***

| **Ranking** | **Question** | **Population** | **Approach** | **Addressed Condition** | **AVG RPS** | **Answerable** | **Program feasible** | **Public health relevant** | **Equity value** |
| --- | --- | --- | --- | --- | --- | --- | --- | --- | --- |
| 43 | What is the prevalence and drivers of child marriage in different humanitarian contexts? | Adolescents (10-19 y) | Background information | Child marriage | 0.765 | 0.768 | 0.744 | 0.768 | 0.780 |
| 46 | What is the prevalence of mental health disorders among refugee adolescents in settlement camps? | Adolescents (10-19 y) | Background information | Mental health | 0.765 | 0.784 | 0.755 | 0.784 | 0.735 |
| 67 | *What are the factors determining health seeking behavior of adolescents in a humanitarian setting? | Adolescents (10-19 y) | Health system strengthening | Crosscutting | 0.735 | 0.775 | 0.706 | 0.735 | 0.725 |
| 73 | In humanitarian crisis, what is the effectiveness of child marriage programming on the mental health and psychosocial wellbeing of adolescent girls? | Adolescents (10-19 y) | Health promotion | Mental health | 0.729 | 0.683 | 0.707 | 0.756 | 0.768 |
| 92 | Does engagement of adolescent peer groups in health response during an emergency improve their health and social outcomes and mental health? | Adolescents (10-19 y) | Health promotion | Mental health | 0.718 | 0.745 | 0.716 | 0.706 | 0.706 |
| 138 | Can the assistance of a team of midwives increase the detection of cases of gender violence in adolescent women in a humanitarian emergency situation? | Adolescents (10-19 y) | Health promotion | GBV | 0.681 | 0.696 | 0.657 | 0.676 | 0.696 |
| 139 | What interventions effectively reduce violence against adolescent females and males in humanitarian settings? | Adolescents (10-19 y) | Health promotion | Violence, injuries, trauma | 0.681 | 0.667 | 0.637 | 0.706 | 0.716 |
| 144 | What are the physical and mental health impacts of forced/child/early marriage of adolescents living in temporary settlements during a protracted humanitarian emergency? | Adolescents (10-19 y) | Background information | Mental health | 0.677 | 0.683 | 0.622 | 0.683 | 0.720 |
| 153 | What kind of mental health assessments and management of disorders among HIV infected adolescents in refugee settlement camps needed to improve their adherence to antiretroviral therapy? | Adolescents (10-19 y) | Health system strengthening | Mental health, HIV | 0.671 | 0.634 | 0.671 | 0.683 | 0.695 |
| 164 | Can facilitated youth groups be used to assess and address the psychosocial needs of adolescents after an emergency? | Adolescents (10-19 y) | Health promotion | Mental health | 0.659 | 0.696 | 0.647 | 0.657 | 0.637 |

**Supplementary Table D. Members, Institutional Affiliations and Terms of Reference of the Technical Advisory Group**

| **Name** | **Organization** | **Email** |
| --- | --- | --- |
| Abir Shady | WHO | shadya@who.int |
| Bernadette Daelmans | WHO | daelmansb@who.int |
| Rudi J. J. M. Coninx | WHO | coninxr@who.int |
| Daniel Martinez | MSF | Daniel.MARTINEZ@geneva.msf.org |
| Gary Darmstadt | Stanford | gdarmsta@stanford.edu |
| Juliet Whitley | DFID | j-whitley@dfid.gov.uk |
| Karl Blanchet | LSHTM | Karl.Blanchet@lshtm.ac.uk |
| Laura Archer | Red cross | Laura.Archer@redcross.ca |
| Lisa Thomas | Red Cross | lisa.thomas@ifrc.org. |
| Olivier Hagon | HCUGE | Olivier.Hagon@hcuge.ch |
| Sachiyo Yoshida | WHO | yoshidas@who.int |
| Tarun Dua | WHO | duat@who.int |
| Zita Weise Prinzo | WHO | weiseprinzoz@who.int |

***The terms of reference of the members of the Technical Advisory group included:***

- Revise, edit and comment on the research protocol and survey tools;
- Provide suggestions and nominate experts for the research prioritization surveys (the first one on soliciting research questions and the second one on the scoring the refined an grouped questions per the different SRMNCAH domains);
- Provide final feedback on the top 10 research priority questions per each of the SRHMNCAH domains;
- Recommend experts for participation in the April 2019 expert group meeting, which was perceived would to minimize selection bias of relevant experts;
- Provide feedback on the expert consultation meeting report before dissemination to participating experts; and
- Contribute to the write up of this manuscript for publication in a peer-reviewed international journal describing the process and advocating for the prioritized SRMNCAH research questions in humanitarian settings.
