# Supplementary material for: Setting research priorities for sexual, reproductive, maternal, newborn, child and adolescent health in humanitarian settings
Source: Confl Health. 2021 Mar 26;15:16. doi: 10.1186/s13031-021-00353-w (PMC7995567; doi:10.1186/s13031-021-00353-w)
Supplement: Supplementary file 2 — Additional file 2: Supplementary Material 1. Interview Guide. Supplementary Material 2. Table 1. Quality criteria for reporting qualitative research (COREQ) and Table 1. Domains, items, description, information in the study. [file 13031_2021_353_MOESM2_ESM.docx]

**Supplementary Material**

**Supplementary Material 1. Interview Guide.**

**Key questions**

Which words do you use in Arabic to describe substance use?

Do you know someone who uses substances? Would you tell us about this person?

What is your understanding of substance use?

What differences do you recognize between substance use and addiction?

Which substances are addictive?

How do you know someone who is addicted?

How is substance use viewed in your community?

What do you think, how does substance use affect everyday life?

How does substance use affect families?

How does substance use affect relationships?

How is substance use developing? What are the factors influencing substance use?

What role does the flight play? When is the first contact with substances usually made?

Are there certain characteristics that indicate the use of (a) substance(s)?

**Supplementary Material 2.**

**Table 1.**

**Quality criteria for reporting qualitative research (COREQ) and**

**Table 1. Domains, items, description, information in the study**

| **No** | **Item** | **Description** | **Information in the study** |
| --- | --- | --- | --- |
| **Domain 1: Research team and reflexivity** | | | |
| ***Personal characteristics*** | | | |
| *Researchers* | | | |
| 1 | Interviewer/facilitator | „Which author/s conducted the interview or focus group?“ | UN *(Methods)* |
| 2 | Credentials | „What were the researcher's credentials? E.g. PhD, MD“ | UN – PhDs, MN doctoral researcher, JL professor, IS – professor |
| 3 | Occupation | „What was their occupation at the time of the study?“ | Research associates, professors |
| 4 | Gender | „Was the researcher male or female?““ | Female and male |
| 5 | Experience and academic training | What experience or academic training did the researcher have?“ | Professionals in the field of mental health research (MPH, medical anthropogist, psychiatrist) |
|  | *Relationship with participants* |  |  |
| 6 | Relationships with participants | Was a relationship with participants established prior to the study? | No |
| 7 | Participant knowledge of the interviewer | Were participants informed about study goals and interviewers? (reasons for doing the research) | Yes, Background, aims, and content of the study *(Methods)* |
| 8 | Interviewer characteristics | What characteristics were reported about the interviewer/facilitator? | Occupation, description of study aims (*Introduction, Methods)* |
|  | | | |
| **Domain 2: Study design** | | | |
| ***Theoretical framework*** | | | |
| 9 | Methodological orientation and theory | What methodological orientation was stated to underpin the study? (e.g., Grounded Theory, ethnocgraphy, phenomenology, discourse Analysis, ethnography, content analysis) | Content- analysis (*Methods, Data analysis)* |
| ***Participant selection*** | | | |
| 10 | Sampling | How were participants selected? e.g. purposive, convenience, consecutive, snowball | Comprehensive sampling strategy *(Methods)* |
| 11 | Method of approach | How were participants approached? e.g. face-to-face, telephone, mail, email | Telepfone, email, face-to-face *(Methods)* |
| 12 | Sample size | How many participants were in the study? | *N*=19 (*Methods, Participants)* |
| 13 | Non-participation | How many people refused to participate or dropped out? Reasons? | Because we used a cosnecutive purposive recruitement strategy we do not have information on drop-outs. |
| ***Setting*** | | | |
| 14 | Setting of data collection | Where was the data collected? (e.g. home, clinic, workplace) | University rooms, rooms of psychosocial institutions *(Methods)* |
| 15 | Presence of non-participants | Was anyone else present besides the participants and researchers? | Yes, interpreter *(Methods)* |
| 16 | Description of sample | What are the important characteristics of the sample? e.g. demographic data, date | Due to confidentaility reasons we did only assess age and have no further personal information (*Methods, Participants).* |
| ***Data collection*** | | | |
| 17 | Interview guide | Were questions, prompts, guides provided by the authors? Was it pilot tested? | Guide was not pilot tested *(Methods)* |
| 18 | Repeated interviews | Were repeat interviews carried out? If yes, how many? | 5 focus group discussions *(Methods)* |
| 19 | Audio/visual recording | Did the research use audio or visual recording to collect the data? | Audio-recordings *(Methods, Procedure)* |
| 20 | Field notes | Were field notes made during and/or after the interview or focus group? | Yes *Methods, Procedure)*. |
| 21 | Duration | What was the duration of the interviews or focus group? | 60–90 min *(Methods, Procedure)* |
| 22 | Data saturation | Was data saturation discussed? | Yes *(Methods)* |
| 23 | Transcripts returned | Were transcripts returned to participants for comment and/or correction? | No |
|  | | | |
| **Domain 3: Analysis and findings** | | | |
| ***Data analysis*** | | | |
| 24 | Number of data coders | How many data coders coded the data? | 2 coders *(Methods, Data analysis)* |
| 25 | Description of the coding tree | Did authors provide a description of the coding tree? | The full analysis with all codes is described within the paper *(Results)* |
| 26 | Derivation of themes | Were themes identified in advance or derived from the data? | Themes were identifed from the data *(Methods, Data analysis)* |
| 27 | Software | What software, if applicable, was used to manage the data? | MAXQDA Version 18 *(Methods, Data analysis)* |
| 28 | Participant checking | Did participants provide feedback on the findings? | No, data was anonymized by time of transcription. *Methods, Procedure)* |
| ***Reporting*** | | | |
| 29 | Quotations presented | Were participant quotations presented to illustrate the themes/findings? Was each quotation identified? e.g. participant number | Yes *(Results),* participants were not identified. |
| 30 | Data and findings consistent | Was there consistency between the data presented and the findings? | Yes *(Results).* |
| 31 | Clarity of major themes | Were major themes presented in the findings? | Our results present the major themes (*Results).* |
| 32 | Clarity of minor themes | Is there a description of diverse cases or discussion of minor themes? | Diverse statements and minor themes are reported (Results). |

Based on: Tong A, Sainsbury P, Craig J. Consolidated criteria for reporting qualitative research (COREQ): a 32-item checklist for interviews and focus groups. International Journal for Quality in Health Care. 2007;19,6:349–57.
